# High-clockrate free-space optical in-memory computing

**DOI:** 10.1038/s41377-026-02206-8

**Published:** 2026-02-13

**Authors:** Yuanhao Liang, James Wang, Kaiwen Xue, Xinyi Ren, Ran Yin, Shaoyuan Ou, Lian Zhou, Yuan Li, Tobias Heuser, Niels Heermeier, Ian Christen, James A. Lott, Stephan Reitzenstein, Mengjie Yu, Zaijun Chen

**Affiliations:** 1 Department of Electrical Engineering and Computing Sciences, University of California, Berkeley, CA 94720 USA; 2 Ming Hsieh Department of Electrical and Computer Engineering, University of Southern California, Los Angeles, CA 90089 USA; 3 Institut für Physik und Astronomie, Technische Universität Berlin, Berlin, Germany

**Keywords:** Optoelectronic devices and components, Imaging and sensing

## Abstract

The ability to process and act on data in real time is increasingly critical for applications ranging from autonomous vehicles, three-dimensional environmental sensing, and remote robotics. However, the deployment of deep neural networks (DNNs) in edge devices is hindered by the lack of energy-efficient scalable computing hardware. Here, we introduce a fanout spatial time-of-flight optical neural network (FAST-ONN) that calculates billions of convolutions per second with ultralow latency and power consumption. This is enabled by the combination of high-speed dense arrays of vertical-cavity surface-emitting lasers (VCSELs) for input modulation with spatial light modulators of high pixel counts for in-memory weighting. In a three-dimensional optical system, parallel differential readout allows signed weight values for accurate inference in a single shot. The performance is benchmarked with feature extraction in You-Only-Look-Once (YOLO) for convolution at 100 million frames per second (MFPS), and in-system backward propagation training with photonic reprogrammability. The VCSEL transmitters are implementable in any free-space optical computing systems to improve the clockrate to over gigahertz, where the high scalability in device counts and channel parallelism enables a new avenue to scale up free space computing hardware.

## Abstract (web summary)

We demonstrated high-speed VCSEL in-memory neural networks that deliver billion optical convolutions per second for massively parallel edge intelligence at ultralow energy and latency.

## Graphical abstract

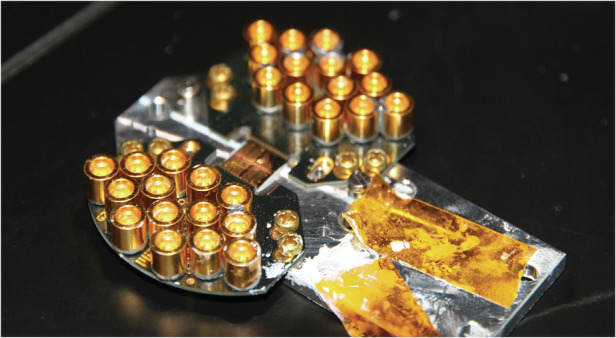

## Introduction

**Fig. 1:**
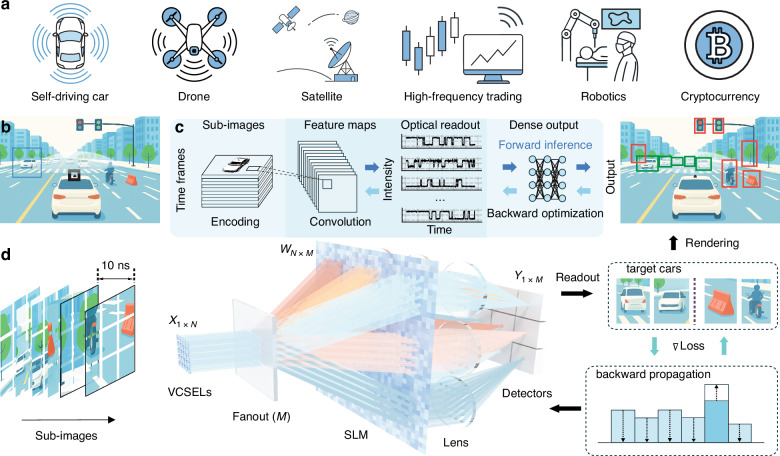
FAST-ONN concept. **a** Example edge-computing applications, including autonomous vehicles, drones, satellites, high-speed trading, robotics, and cryptocurrency. These scenarios span mobile, embedded, and high-frequency decision-making environments, where low-latency and energy-efficient AI inference is critical. **b** Application scene. A car in the left-forward quadrant is detected and highlighted with a bounding box as the region of interest (ROI). This ROI is then routed into the dataflow for optical inference in (**c**). **c** An edge camera catches images and processes them with parallel convolution to generate multiple feature maps, which yield multichannel optical readout signals feeding downstream outputs. Forward inference and backward optimization are both supported, enabling in-situ training. **d** Sub-images are encoded onto a VCSEL array of *N* elements and fanned out into *M* parallel copies, where convolutional layers are executed on FAST-ONN; a trained model is loaded for inference, and the readout can be fed back to update weights for in-line parameter optimization. The resulting detections are rendered back onto the original scene, with cars outlined in green and other objects in red

The emergence of artificial intelligence models is revolutionizing information processing with high precision in perception, reasoning, and control^1^. Deploying these AI models to the edge devices enables smart sensors with information processed directly where data is generated, ensuring real-time responsiveness, reducing latency, and minimizing energy-intensive cloud-edge data transfers. However, edge devices operate in environments that are flooded with dynamic and complex information, such as three-dimensional (3D) sensing in autonomous driving^2,3^, defense warfield^4,5^, or remote robotics^6,7^ (Fig. 1a). Processing this information can require powerful models with enormous size and complexity that are difficult to deploy on edge platforms, due to the limited computing power, compact form factors, and strict energy budgets^8,9^, which are stretching the performance of existing processors. The heart of deep neural network (DNN) models lies in large-scale matrix-vector multiplication (MVM), which requires intensive data parallelism, where conventional central processing units suffer from the Von Neumann bottleneck, and state-of-the-art accelerated computing processors, including graphics processing units^10^, tensor processing units^11^, field-programmable gate arrays^12^, and application-specific integrated circuits^13^, allow to rely on the movement and storage of charges with electronic wires that lead to low clock rates and high thermal dissipation due to capacitive losses^14–16^. Scaling up the computing power with complementary metal-oxide semiconductor platforms further increases size-, weight- and power- (SWaP) constraints^17,18^. For example, many edge-deployed sensors, such as light- or microwave-based ranging devices and cameras on autonomous vehicles^19^, incur substantial power draw and thermal load merely to capture and pre-process raw data. This energy expenditure not only reduces operational range but also necessitates active cooling, compounding overall system power consumption and undermining reliability in mobile or remote deployments^20,21^. Therefore, performing high-speed, large-scale, and low latency matrix operations under strict SWaP constraints remains a key bottleneck for edge AI hardware.

Photonic solutions are emerging to accelerate computation of matrix algebra with advantages of ultrahigh clockrates, low-loss propagation and high parallelism. Recent progress on photonic integrated platforms^22–25^ using cascaded Mach-Zehnder interferometers (MZIs^22–24^), optoelectronic memristive device^26^, wavelength multiplexed weight banks^27^, parallel operations with phase charge materials^28,29^ and thin-film lithium niobate photonics^30–32^ have achieved high degrees of integration and programmability. Remarkable efforts have scaled up these systems to thousands of devices^33,34^, but further scaling is limited due to the large device footprints, limited chip areas, fabrication errors, and control and packaging complexity. Alternatively, implementing free-space systems based on light propagation allows promising system scaling and massive parallelism within the aberration limit. Besides, weighting with 3D-printed diffractive elements^35,36^ or spatial light modulators (SLMs) provide millions of individually controllable weights^37,38^. However, the computational clock rates relying on the input data transmitters, using 3D-printed images^39,40^, digital micromirror devices^41,42^, SLMs^43,44^ and organic light-emitting diodes^37,45^ are typically slow (less than 100 kHz), and this has been improved in a recent breakthrough with micro light-emitting diodes that improved the clock rate to the megahertz range^46^. Therefore, the system throughput is limited by the low clockrates. To fully unlock the potential of light, here we propose FAST-ONN to speed up these systems, where it explores (1) densely-packed volume-manufactured VCSEL transmitter arrays for input activation; (2) spatial fanout coping with diffractive element for parallel processing; (3) large-scale programmable weighting for in-system training; (4) low latency image classification under the You-Only-Look-Once (YOLO)^47^ algorithm to provide accurate feature extraction.

## Results

The FAST-ONN is efficient for general matrix multiplication (GEMM). FAST-ONN consists of an input encoding layer, a spatial fanout layer, a weighting layer, and a read-out photodetector (PD) array. Here, each subimage with *N* pixels (Fig. 1d) *X*_(*N*×1)_ is encoded to an array of *N* VCSEL devices. The emitted optical signals from the VCSELs are replicated to *M* copies through a diffractive optical element (DOE). Each multiplies a weight kernel consisting of *N* pixels on a SLM. This element-wise multiplication is completed with light propagation through cascaded modulation, and the accumulation occurs by summing the intensity of the *N* beams onto a photodetector, which generates photocurrents *Y*_*M*_ = *I*(*m*) ∝ ∑_*N*_*W*_*m,n*_*X*_*n*_, where *n* and *m* denote the input pixel index and the fanout copy index, respectively. At each clock cycle, *M* high-speed detectors read out the matrix-vector products of *M* kernels simultaneously. The signals captured by the detectors are digitized and processed through downstream neural layers and a classification head, which generates the recognition result for each subimage. In contrast to our previous approach of VCSELs in homodyne time-multiplexed coherent photoelectric multiplication^48^, the FAST-ONN achieves multiply-accumulates with cascaded modulation that does not require precise coherent control or phase-amplitude coupling that limits the computing accuracy, while preserving the system simplicity and scalability.

To benchmark its performance given the limited driver electronics, we implemented convolutional operations, which account for the vast majority of the total computational cost, often exceeding 70% in well-known algorithms like Visual Geometry Group (VGG)^49^, ResNet^50^, and YOLO^47,51^. In a convolutional neural network (CNN), kernels slide across the image, performing local multiplications with subimages to extract feature maps. We deliberately employ kernels of 3 × 3 pixels for all benchmarking tasks, as this convolution kernel size is favored in modern CNNs (e.g., VGG^49^, U-Net^52^, and ResNet^50^) for its strong representational power and computational efficiency, while also fitting our hardware’s capacity and bandwidth. Parallel operations in FAST-ONN allow simultaneous processing of multiple kernels at high clock rates. These computing results are rendered back to reconstruct the full scene, with task-specific labels (such as cars or non-car objects). Benefiting from the reconfigurability of the SLM, the system output can be utilized as feedback to update the spatial weighting, thereby enabling in-system parameter updates and real-time training tailored for different application scenarios such as self-driving cars, drones, and robotics (Fig. 1a, b).

**Fig. 2:**
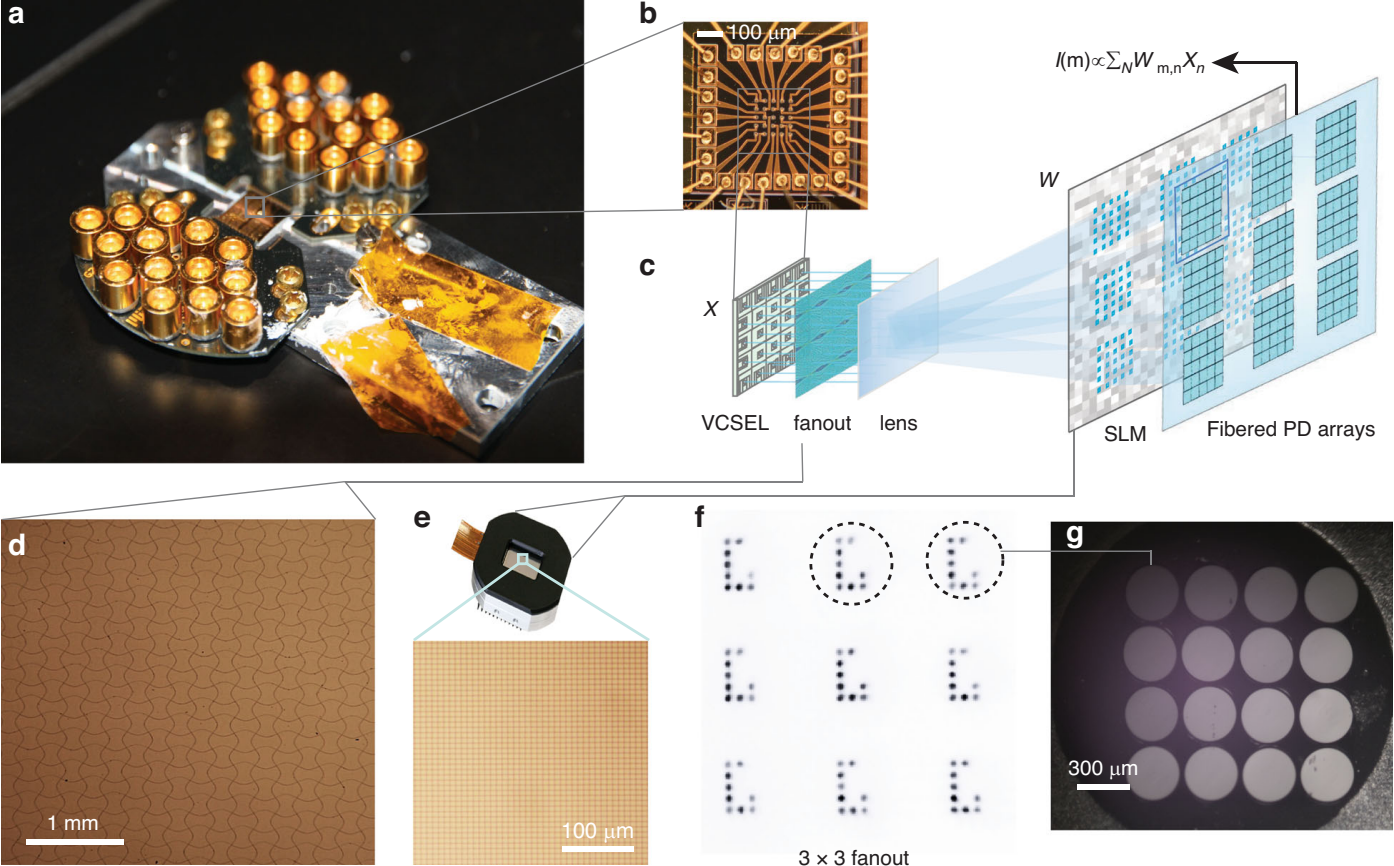
Experimental demonstration of FAST-ONN. **a** Fabricated arrays of VCSELs implemented in our FAST-ONN system with eight arrays each with 5 × 5 VCSELs. **b** Zoomed-in view of an array of 5 × 5 VCSEL used in the experiment. **c** Schematic of the FAST-ONN. The system comprises a VCSEL array that emits input-encoded optical signals, which are fanned out into multiple spatial copies via a DOE. These separated fanout beams are then modulated in parallel by a SLM to apply the desired convolution kernel weights. The weighted optical signals from each copy are summed by a multimode fiber with a core diameter of 300 μm and detected by a set of balanced photodetectors (BPD). **d** Microscope image of the phase pattern encoded in the DOE used for 3 × 3 fanout. **e** SLM and a zoomed-in view showing the individually addressable liquid crystal pixels. **f** Experimental 3 × 3 fanout pattern of VCSEL array encoding a letter “C”. **g** Two-dimensional fiber array implemented in the system, one copy in the weighted arm and one in the reference arm

Our FAST-ONN is developed with a VCSEL chip of eight arrays, each with 5 × 5 VCSELs (see “Methods” for details on the fabrication). The electrical contacts of the VCSELs are routed with short wires to maintain the bandwidth after wire bonding. In contrast to the linear array^46^, our square array geometry supports both higher device counts and higher fanout factors at the aberration limit^44^, as discussed in the Supplementary Information. Given the compact device size, a total of 400 VCSELs are fabricated in the chip area of about 10 × 10 mm^2^ (Fig. 2a). The modulation bandwidth of the VCSELs is characterized to be 1 GHz (measured in^48^), limited in the present device design by the cavity lifetime. Noting that state-of-the-art VCSELs have achieved a bandwidth over 45 GHz^53^. Here, all the VCSELs are designed specifically with a slightly-elliptical cavity shape to emit linearly polarized light around 973 nm, which is important for efficient intensity modulation with the SLMs (Methods). In our experiments, we use a single array with 25 VCSELs (Fig. 2b). While a single VCSEL can emit a maximum power of ~6 mW, we experimentally set them to about 2 mW per device to achieve our desired signal-to-noise ratio. The beams from the 5 × 5 array are fanned out to 3 × 3 copies using a diffractive optical element (Fig. 2d). For example, Fig. 2f shows the resulting beams when encoding a letter “C”. The average ellipticity of all the fanout beam spots is 1.004 (≈1), and the full width at half maximum (FWHM) has a 1.3% coefficient of variation, indicating uniform spot shape with minimal aberration. Each copy with 5 × 5 beams is weighted on the SLM (Fig. 2e, f) and collected with a multimode fiber array (Fig. 2g) connected to an array of high-speed detectors.

**Fig. 3:**
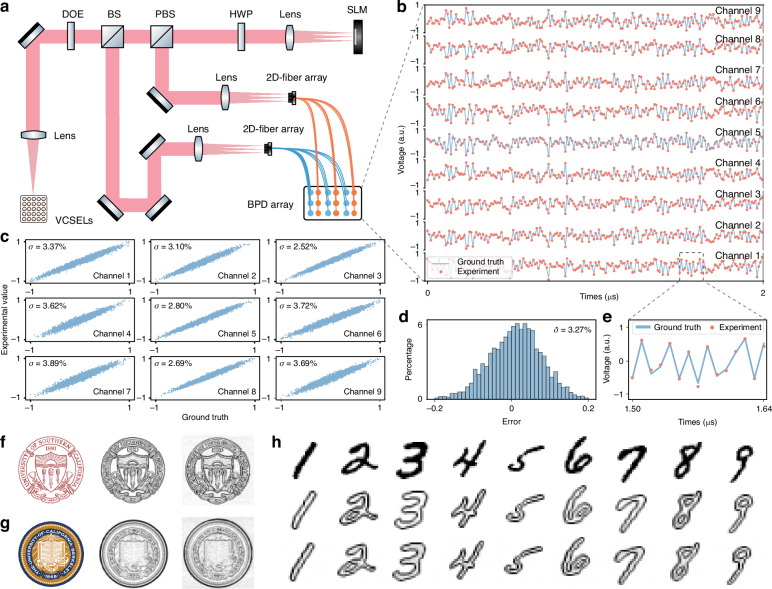
Simultaneous acquisition of random MVM with FAST-ONN. **a** FAST-ONN experimental setup. DOE diffractive optical element, BS beam splitter, PBS polarized beam splitter, HWP half waveplate, SLM spatial light modulator. **b** Comparison of experimental results and digital ground truth for the multiplication of a randomly distributed input vector with signed weights. The input is encoded onto a 9-VCSEL array operating at 100 MS s^−1^, while the weights are encoded on the SLM. **c** Correlation between ground truth and experimental outputs from the 9 optical channels. **d** Histogram of multiplication errors across all optical channels, showing an average error of 3.27%. **e** Enlarged view of experimental results versus ground truth. **f**, **g** Edge detection results of the University of Southern California logo (**f**), the University of California, Berkeley logo (**g**). **h** Edge detection results of handwritten digits from the MNIST dataset. The top, middle, and bottom rows show the original data, digital ground truth, and FAST-ONN detected edges, respectively

A key challenge for intensity-based optical computing is encoding signed weight values, which are essential for neural network accuracy. FAST-ONN addresses this by using parallel reference beam and differential photodetection. All the VCSELs are set to be mutually incoherent to avoid any undesirable interference. To implement signed weights, the VCSEL beams after the fanout DOE are split into two paths (Fig. 3): the signal beams are directed to the SLM for polarization-based weight modulation (Methods), and the other path serves as reference beams. The beam copies in the signal beams and the reference beams, respectively, are separately coupled into 2‑D fiber arrays, which deliver the beams to the positive and negative ports of the 3 × 3 balanced photodetectors (BPD) that perform differential photocurrent subtraction. All 3 × 3 signal and reference beams are collected with 300-µm core diameter multimode fibers and digitized through a 9-channel FPGA-based data acquisition board at 100 MS s^−1^. This parallel non-interferometric differential readout extends FAST-ONN beyond unsigned intensity accumulation for general signed-weight convolutional tasks.

### Parallel computing accuracy

We verify the computing accuracy in FAST-ONN by encoding a set of signed randomly distributed numbers at a rate of 100 MS s^−1^ to the array of nine VCSELs (*N* = 9). The 3 × 3 copies of the VCSEL beams are weighted with the SLM pixels of normally distributed randomly assigned values. The experimental time traces, each with a duration of 2 μs and with 200 multiply-accumulate (MAC) operations across all nine output channels, exhibited good agreement with numerical calculations (Fig. 3b–e). Analysis of the residuals |y−ŷ| revealed an average computation error standard deviation of 3.27% across nine channels. We attribute the error to the VCSEL encoding linearity due to the impedance mismatch of the drivers and the summing errors, as analyzed in Supplementary Figs. S8 and S9. This accuracy over 6 bits (5 bits for the numerical accuracy and 1 bit for the sign) is sufficient for most neural network tasks^54^. In image processing tasks, FAST-ONN allows real-time convolution of arbitrary input images. As an example, the university logos and handwritten digits are vectorized and encoded onto the VCSELs (Fig. 3f–h) at 100 MS s^−1^. Applying an edge detection kernel on the SLM allows revealing the target information. The resulting edges are consistent with ground-truth with over 95.6–96.3% accuracy, which corresponds to about 5–6 bits accuracy. This is about 1–2 bits higher than other state-of-the-art free-space optical computing systems^37,44^, as detailed in the Supplementary Table S2.

### You only look once

Object-level car classification is a stringent, deployment-relevant benchmark for FAST-ONN. It matches YOLO-style tasks in autonomous driving and is central to intelligent transportation^55^, where reliable car identification supports decision-making and safety^56^. Meeting these edge demands calls for fast, scalable, and robust semantic processing that strains conventional digital DNNs^57^. Here we benchmark FAST-ONN in accelerating YOLO tasks while maintaining accuracy. To test under realistic visual complexity, we adopt Common Objects in Context (COCO) dataset^58^, as it provides diverse, fine-grained objects in cluttered scenes and is widely used for detection and classification in real-world settings.

**Fig. 4:**
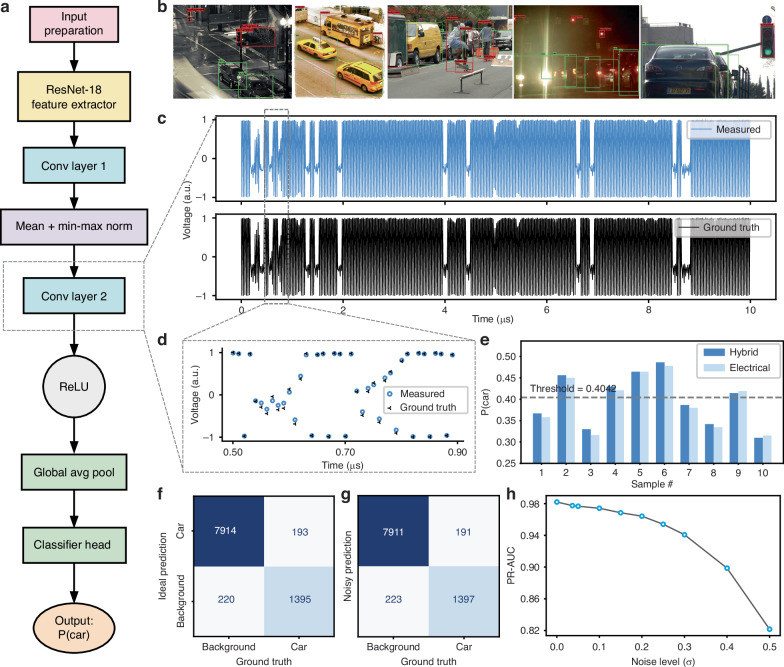
Object-level car classification and convolutional noise analysis using FAST-ONN. The system evaluates whether a detected object corresponds to a car using a hybrid pipeline. **a** Workflow of the object-level classification algorithm applied to the bounding-box-cropped images. **b** Inference results with green boxes indicating objects identified as cars and red boxes denoting background. **c** Time-trace of the layer-2 convolutional output from FAST-ONN compared with the electrical reference, with the error of 0.037. **d** Zoom-in of a segment from (**c**), showing good consistency with the ground truth. **e** Comparison of final classification probabilities with and without FAST-ONN integration, with a trained decision threshold of 0.4042. **f** Confusion matrix of the full classification pipeline under ideal conditions. **g** Confusion matrix obtained with experimental noise applied to all convolutional layers, including ResNet-18 layers 1–2 and Convolutional layers 1–2. **h** AUC versus the injected noise level, ranging from *σ* = 0 to *σ* = 0.5

We constructed an object-level car-vs-background classifier on COCO with ResNet-18 backbone to supply realistic inputs for evaluating FAST-ONN (Fig. 4a). Bounding-box-cropped patches are curated and resized under consistent quality and size constraints, and ResNet-18 is fine-tuned on COCO by unfreezing the last two residual stages and appending two lightweight convolutional layers to sharpen local features. The pipeline achieves accurate object-level classification, producing predictions with green bounding boxes indicating cars and red boxes indicating background (Fig. 4b), and attains an area under the receiver operating characteristic (ROC) curve (AUC) of 0.98, demonstrating its effectiveness in handling challenging, cluttered real-world scenes. In the experiment, we apply the second convolution layer with 2 × 2 kernels, in the hardware system. All subsequent operations, including rectified linear unit (ReLU), global average pooling, and the classification head, are digitally executed. Although it’s not fully computed in the optical chip, this model verifies our computing accuracy is sufficient to support complex operation tasks that require real-time decision making and all the layers may be implemented in the FAST-ONN when the system scales up. Using 2000 randomly selected test samples from COCO, we compared the outputs computed by the hybrid optical-electrical system with those from the purely electrical baseline. The two systems showed excellent agreement, with a standard deviation of 0.037 between outputs (Fig. 4c, d). Fig. 4e shows the probability distributions of car classification, which were obtained by feeding the outputs from the FAST-ONN and pure electrical inference into the convolutional layer of the system. Robustness under Gaussian noise was evaluated to simulate system performance under varying hardware imperfections (Methods). The performance gradually declines as the perturbation variance increases, yet remains stable with an AUC above 0.82 even at *σ* = 0.5, indicating a strong tolerance for hardware defects under the expected operating conditions.

### CNN inference and training

**Fig. 5:**
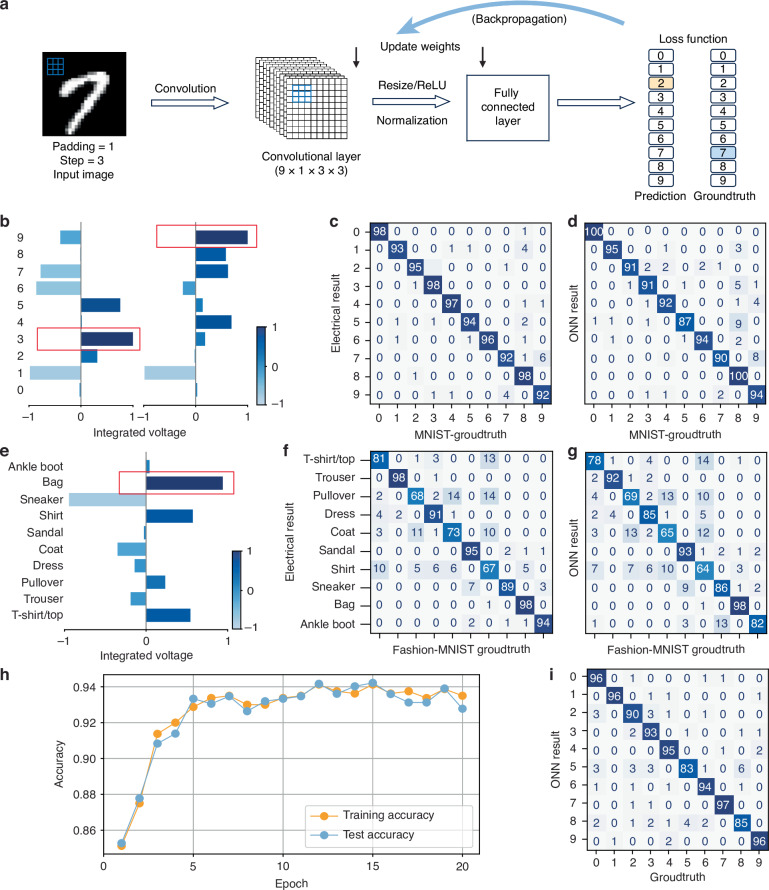
Benchmarking of machine learning inference and in-system training with FAST-ONN. **a** Neural network model for MNIST and Fashion-MNIST classification. The model consists of one convolutional layer with nine 3 × 3 kernels, followed by a fully connected output layer. Forward propagation results are compared against ground truth to compute the loss and update both the convolutional kernels and output weights. Output layer readout for MNIST (**b**) and Fashion-MNIST (**e**). The final classification is determined by selecting the label with the highest output value. Comparison of electrical (**c**) and optical (**d**) confusion matrices for MNIST classification with 800 randomly selected from test dataset (electrical 95.75%, optical 93.75%). Comparison of electrical (**f**) and optical (**g**) confusion matrices for Fashion-MNIST classification with 800 randomly selected from test dataset (electrical 84.88%, optical 80.75%). Optical and electronic results exhibit close agreement, validating the inference capability of FAST-ONN. **h** In-line training accuracy versus epoch (20 epochs: 93.5% train subset, 92.8% test subset). **i** Confusion matrix after 20 epochs of in-system training

To benchmark its performance, FAST-ONN was evaluated on the standard 10-class Modified National Institute of Standards and Technology (MNIST) and Fashion-MNIST datasets. The network architecture used for both tasks is shown in Fig. 5a and consists of three main stages: an input layer, a convolutional layer, and a fully connected classification head. The input layer reshapes each 28 × 28 image into 10 × 10 subimages of 3 × 3 pixels, which are encoded and transmitted optically using 3 × 3 VCSEL arrays. The convolutional layer performs fanout, multiplication, and accumulation using 9 distinct 3 × 3 kernels; this stage is fully executed in the optical domain. Finally, the fully connected layer maps the resulting 900-dimensional vector to 10 output classes digitally, providing the final classification results.

We train the digital reference on the full MNIST and Fashion-MNIST training sets using sparse categorical cross-entropy for 10 epochs, and evaluate on the held-out tests (Fig. 5b–g; training details are demonstrated in “Methods”). The digital model attains 95.75% on MNIST and 84.88% on Fashion-MNIST, while hardware inference achieves 93.75% and 80.75% respectively, closely matching the digital baseline.

In addition to real-time inference, being able to adapt rapidly to the diverse environmental changes is a necessity for edge intelligence, but it requires on-device, in-line training^23,59^, to avoid long-haul data transfers and to reduce latency and energy overhead in scenarios such as autonomous vehicles, drones and real-time robotics. To realize this training in FAST-ONN, we couple optical forward passes at 100 MS s^−1^ with digital gradient updates, training on 800 randomly selected images from the MNIST training split (fixed seed) and evaluating on an independent 800-image subset from the test split. Inputs are forwarded through the optical convolution, cross-entropy loss is formed from digitized readouts, and weights are updated by gradient-based optimization under an element-wise [−1, 1] constraint. We computed the gradients digitally with ReLU gating and backpropagated through the sampled 3 × 3 patch structure while the forward path streams. After 20 epochs, the system reaches 93.5% accuracy on the training subset and 92.8% on the test subset (Fig. 5h, i), demonstrating practical on-device learning within the live inference path.

## Discussion

### Throughput

We experimentally achieved optical convolution with a 5 × 5 VCSEL array in 3 × 3 parallel kernels at 100 mega subimages per second, corresponding to 0.9 billion convolutions per second in both training and inference tasks. Its performance is benchmarked with over 98% computing accuracy in a COCO classifier for inference of YOLO tasks, and in-line training illustrates the hardware reprogrammability. The computational throughput in FAST-ONN *T* = 2 × *N* × *M* × *R* increases with VCSEL device counts (*N* = 5 × 5) and fanout factors (*M* = 3 × 3). Currently the throughput *T* = 45 GOPS, limited by the low channel counts and clockspeed *R* = 100 MS s^−1^. Based on the volume-manufactured device platforms and the large-scale spatial parallelism, a system with 32 × 32 VCSELs and 32 × 32 fanout factors may be achieved in the near term. As the detailed analysis in the Supplementary Information discusses, these high channel counts are achievable with our compactly-arrayed VCSEL devices to enable large-scale fanout and make use of the high number (>1 million) of SLM weighting pixels available. However, scaling to a larger fanout factor may introduce challenges such as potential optical crosstalk between beams on the SLM and aberration control across the wide field of view. Addressing this requires coupling optics with a higher numerical aperture (NA) and robust aberration control, as detailed in the Supplementary Information.

With a VCSEL clock rate of 25 GS s^−1^ (VCSEL bandwidth over 45 GHz has been demonstrated^53^), a total throughput *T* > 50,000 TOPS may become achievable, which would be over 10× the performance of an electronic system (4000 TOPS in NVIDIA H100^60^).

### SLM refresh limits

An important application scenario for FAST-ONN is low-power AI inference at the edge, where low latency is critical and frequent weight updates are unnecessary. In such scenarios, the system’s overall computation speed is primarily limited by the clock rate of the VCSELs, rather than the refresh rate of the SLM. For example, a pre-trained model can be loaded onto the SLM in advance, allowing high-speed computation without further updates during execution. Although the liquid-crystal SLM operates at a relatively slow refresh rate (~100 Hz), it is sufficient for adapting to in-situ environmental changes without affecting inference throughput under fixed weights. While the limited SLM speed may constrain applications that require frequent weight updates, such as backpropagation during training, it might be less of a bottleneck in practice. In typical training workflows, all input data are processed with the same weights (O(*N*²) operations) before gradients are computed (O(*N*) operations). For small datasets, SLM update speed may become a limiting factor; however, for most practical training tasks involving large datasets, the time needed to compute across all inputs might be longer than the SLM’s 10 ms refresh time, allowing FAST-ONN to operate at full throughput even in training contexts.

### System latency

We quantified the end-to-end delay by considering input encoding, light propagation, photodetection, digitization, and digital post-processing. To compute 9 kernels of 3 × 3 VCSELs, encoding at 100 MS s^−1^ takes 10 ns per image, propagation over 1 m in the setup in air takes about 3.3 ns and the readout takes 10 ns with the analog-to-digital converter (ADC), this totals to about 23.3 ns to process a single subimage with 9 kernels.

In the CNN image classification tasks, a 28 × 28 input is padded to 30 × 30 and propagated through a 3 × 3 receptive field (step = 3) over 100 positions; at 100 MS s^−1^, each position takes 10 ns, resulting in a total streaming time of 1.0 µs. The encoding and readout are synchronized and continuous, so the total delay is only limited by this streaming time. The remaining digital computation (19,138 FLOPs) requires ~0.4 µs on a representative processor core, assuming a compute rate of 50 GFLOP s^−1^, resulting in a total input-to-decision delay of ~1.4 µs.

Currently, this latency is primarily affected by the low clock rates and the channel count. However, if the VCSEL modulation and readout rate is increased from 100 MS s^−1^ to 25 GS s^−1^, which decreases the subimage processing time to ~40 ps, thereby greatly reducing the overall transmission time to approximately nanosecond-scale. The fully connected layer can be implemented with high channels. The latency bottleneck shifts from electronic serialization to the optical propagation, which can be further mitigated by a compact system design.

### Scalability

A key requirement for the high fanout channels is sufficient optical power for the readout signal-to-noise ratio to support the target computing bit precision. However, this does not set a constraint in our FAST-ONN, because here the VCSELs serve as both laser sources and transmitters. Scaling up with high channel counts with VCSELs not only increases the data bandwidth, but also increases the total power. Our single-mode VCSELs can emit up to 6 mW, which results in a total power of over 6 W with the 32 × 32 VCSEL channels. The same power level would be challenging to reach with waveguide-based systems^61–64^ due to power-dependent nonlinearities in the waveguides and on-chip lasers. As shown in Supplementary Fig. S7, we provide a detailed analysis regarding the computing bit precision, which suggests that 1 mW optical power on each detector is sufficient to support the signal-to-noise ratio of 7–8 bits computing at a clock rate of 25 GS s^−1^. This only requires about 1 W total optical power for the 32 × 32 parallel channels.

Another key requirement is having O(*N* × *M*) channels of weighting devices, which is achievable with *N* × *M* = 1 million SLM pixels using off-the-shelf components (e.g., our SLM provides 1920 × 1200 = 2,304,000 pixels), and each pixel can be independently modulated and functions as an individual weighting unit^44^. Compared to on-chip integrated circuits, achieving millions of photonic devices (e.g., MZI mesh^22^, memristive crossbar arrays^28,29^, microring weighting banks^65^) is fundamentally challenging. Compared to other free-space systems that operate <10 MHz^35,37,40,43^, FAST-ONN is based on a compact and high-speed VCSEL platform which supports 1000× improvement in clock speeds and dense arrays for high fanout copies.

In addition to scaling through spatial parallelism in fanout factors and VCSEL arrays, increasing the computing dimension, such as via orbital angular momentum modes, also offer an orthogonal and promising route^66^. We also provide several compact designs in Supplementary Fig. S10–S12, which further illustrates the feasibility of integrating the system into a smaller footprint while maintaining scalability.

### Energy efficiency

We analyze the full-system energy consumption, including the electronic peripheral (Supplementary Fig. S2). Each *X*-vector from electronic memories is converted to drive the VCSELs using digital-to-analog converters (DACs). After optical fanout parallel computing, each MAC product is read out with a photodetector, followed by a transimpedance amplifier (TIA) and an ADC. The full-system electrical power, including the VCSEL drivers (bias power for laser generation and DAC drivers), the weighting power on the SLM pixels, and the readout electronics (Supplementary Table S1). With appropriate electronic circuitry, our FAST-ONN system can achieve an energy efficiency of about 370 fJ·OP^−1^ (~3 TOPS·W^−1^), which is similar to a state-of-the-art GPU. The energy cost for electronic-optical-electronic conversion can be further amortized by the high channel parallelism. If the system scales to the 32 × 32 VCSELs with 32 × 32 fanouts, an efficiency of 2 fJ·OP^−1^ (500 TOP·W^−1^) might be reached (Supplementary Table S1), which corresponds to 100× improvement compared to state-of-the-art electronic computing (~5 TOP·W^−1^ in NVIDIA H100^60^). In Supplementary Table S2, we provide a comparative summary of throughput, energy efficiency, device characteristics, and programmability compared to other state-of-the-art optical processors.

### Towards deep neural networks

We envision an optoelectronic multi-layer architecture composed of multiple DAC-VCSEL-DOE-SLM-PDs-ADCs modules (Supplementary Fig. S2) in each neural network layer. In this scheme, the MVM of each layer is performed optically, followed by digitization, nonlinear activation, and re-encoding before being loaded into the next optical layer. This explores the advantages of FAST-ONN for low-energy high-speed matrix multiplication, while keeping the signal integrity and scalability in each layer. For edge inference, this approach does not require frequent updating of the SLMs, as the weights are preset of a given pretrained model. Alternatively, to perform multiple cascaded layers all in the optical domain (Supplementary Fig. S3), the optical signal can be routed to be weighted over multiple cascaded SLMs or reflective passes on a single SLM, where power-dependent nonlinearity can be implemented with an intensifier^45^ or exciton-polariton materials^67–70^.

In conclusion, FAST-ONN combines high-speed, compact VCSEL transmitters with large-scale programmable spatial weighting, enabling reconfigurable and parallel AI computing. The compact, high-speed, fabrication-ready VCSEL platform can be exploited in any free-space optical computing system, including diffractive neural networks and optical generative models^35,71^ and fanout systems^44,45^ to improve the clock rate and thus throughput. FAST-ONN provides strong inference capabilities and supports backward optimization, enabling real-time tasks and has successfully implemented vehicle recognition in a hybrid optoelectronic pipeline. FAST-ONN is positioned to drive a compounding advance in capability and efficiency from edge deployments to large-scale model regimes, charting a more efficient trajectory for computing.

## Materials and methods

### Dataset

The dataset of MNIST handwritten digits, MNIST fashion products, and COCO objects were respectively taken from http://yann.lecun.com/exdb/mnist/ and https://github.com/zalandoresearch/fashion-mnist, and https://cocodataset.org.

### VCSEL fabrication

The VCSEL devices are based on semiconductor heterostructure microresonators, comprising a gain region formed by a stack of InGaAs quantum wells enclosed between two AlGaAs/GaAs distributed Bragg reflectors (DBRs) acting as cavity mirrors^72^. Each VCSEL within the 5 × 5 array was patterned via UV lithography and etched using an inductively coupled plasma reactive-ion process. To achieve high integration density, the array was arranged with a compact pitch of 80 μm between adjacent devices (Fig. 2a). Electrical interfacing was realized by depositing Au-based p-contacts on each cavity, which were individually wire-bonded to a printed circuit board connected to external driving electronics. All VCSELs in the array share a common ground connection to simplify electrical routing. A polymer cladding layer was applied over the chip to enhance mechanical stability, with selective openings above the apertures to allow light emission. The emitted beams exhibit stable linear polarization due to a modest cavity ellipticity, based on the precision of UV lithography. Further details on polarization control are provided in the Supplementary Information.

### VCSEL bandwidth

The modulation bandwidth of the present VCSELs is limited to 2 GHz due to both device‑ and system‑level factors. At the device level, the high cavity Q‑factor (~10⁵) lowers the lasing threshold but increases photon lifetime, restricting the achievable bandwidth^48,53^. Additional resistance-capacitance parasitics in the device structure further attenuate high‑frequency modulation. These effects can be mitigated by reducing the Q‑factor, decreasing the cavity mode volume, and employing Vernier‑effect designs that preserve output power with a large aperture^53^. At the system level, long wire bonds and interconnect traces in the 2D array introduce parasitic inductance and capacitance, which distort high‑speed signals. In future, moving toward back‑emitting VCSELs with flip‑chip bonding would shorten electrical paths, reduce parasitics, and enable significantly higher modulation speeds.

### Experimental setup

The laser output from the VCSEL array is collimated by a converging lens (focal length = 150 mm) and the collimated beam then passes through a DOE (MS-711-K-Y-X, Holo/Or) with a diffraction angle $\theta$ = 0.26 degrees. A beam splitter divides the beams into two beams for differential detection. One beam is focused onto the SLM (Santec Corp., Japan) by a lens with a focal length of 400 mm. The resulting optical spot formed on the SLM has a diameter of 19.7 μm, allowing it to be covered and modulated by a 3 × 3 array of SLM pixels. The center-to-center pitch between adjacent VCSELs, when projected onto the SLM is about 200 μm, and the fanout center distance is around 1800 μm. This ensures that the fanout copies of the whole 5 × 5 VCSEL light spots are well-separated on the SLM (Fig. 2f). Following SLM modulation with a polarizing beam splitter, the optical beams are expanded to increase the center-to-center spacing of each fanout copy to 330 μm in the image plane. This expansion enables one-to-one coupling between individual fanout copies and the corresponding channels of a 2D fiber array (Beijing Reful Co. Ltd.), such that each fiber core receives a distinct spatial copy. An identical expansion is applied to the reference beam path, where a separate 2D fiber array captures the unweight replicas. Both fiber arrays have a common core pitch of 330 μm and a core diameter of 300 μm, ensuring efficient coupling with minimal crosstalk between adjacent channels. Each channel of the two 2D fiber arrays is routed to a balanced photodetector (Koheron, France), such that the two input ports of each BPD receive optical signals from corresponding channels in the modulated and reference paths, respectively. The electrical outputs from the BPD array are subsequently organized and routed through a patch panel, which facilitates stable cable management and flexible signal distribution before being connected to the acquisition card for digitization and processing.

### VCSEL calibration

Although our VCSELs exhibit a good linearity within a defined operating range, the device-to-device input-output variations can introduce errors in power summing. To address this, we perform per-device calibration by first characterizing the injection current range that yields a uniform optical intensity range across all VCSELs. A dedicated look-up table (LUT) is then generated for each VCSEL to linearize the input-current mapping, with a measured linearity of 0.998, ensuring consistent optical output across the array. Details on VCSEL fabrication and long-term stability measurements are discussed in the Supplementary Information.

### SLM calibration

We pre-calibrate the PBS-SLM reflection to ensure that the reflected power is linear in the target weight *W*_*n,m*_. For each macropixel (tile), we measure the reflected intensity as a function of aggregate grayscale level *g*, fit a monotonic curve, and invert it to generate a per-macropixel LUT that maps *W*_*n,m*_ to the required *g*. The linear fit achieves a coefficient of determination of 0.991. To compensate for residual variation across the array, a flat-field gain map is applied to equalize the dynamic range across all fanout channels. Device nonidealities are not embedded in training. Instead, we apply the pre-calibrated macropixel LUT and gain map only during the physical inference, thereby minimizing training overhead and improving overall workflow efficiency.

### Polarization modulation

After calibration, we turn phase modulation into amplitude weights by polarization gating with a PBS-HWP-SLM stack (Fig. 3a). The VCSEL beams pass through the PBS and a HWP rotates their linear polarization to 45 degrees relative to the SLM modulation axis. The SLM introduces a drive-dependent phase difference *φ* between eigenpolarizations; projection onto the PBS reflection port yields *I*_*R*_ ∝ sin^2^(*φ*/2). Using the LUT *g*(*W*_*n,m*_) and flat-field gains from SLM calibration, this response is linearized so the reflected power varies proportionally with *W*_*n,m*_, and all fanout channels share the same linear response.

### Noise robustness

To evaluate the tolerance for overall hardware imperfections in a portable way, we applied an additive zero-mean Gaussian perturbation (variance *σ*^2^) after the activation of every convolutional layer (Resnet-18 backbone and the two task-specific convolutional layers). The evaluation was performed with 2000 test images to ensure statistical reliability. We used *σ* = 0.037 from Fig. 4g and scanned *σ* in [0, 0.5] in Fig. 4h to detect margin. This hardware aligned but device independent perturbation simulates the cumulative effects of PD/TIA/ADC readout noise, VCSEL power fluctuations, and SLM weight perturbations without relying on device specific calibration, enabling homogeneous comparisons between different model variants. Compared to the noise free baseline (Fig. 4f), the performance steadily decreased during the scanning process and maintained AUC above 0.82 at *σ* = 0.5 (Fig. 4h), demonstrating robustness under the intended operating conditions.

### CNN training

We implement a compact convolutional classifier to match our intended hardware mapping. MNIST and Fashion-MNIST images (28 × 28 pixels) are normalized to [0, 1], reshaped to (28, 28, 1), and zero-padded by one pixel on all sides to form a 30 × 30 input. The network’s core is a single bias-free 2D convolutional layer with nine 3 × 3 filters (stride = 3) followed by ReLU. This configuration produces nine 10 × 10 feature maps, which are flattened into a 900-dimensional vector and passed through a bias-free dense layer with softmax activation to yield ten class probabilities. Omitting biases streamlines our downstream optical implementation, and the chosen stride-and-padding scheme ensures that the network’s spatial sampling aligns exactly with our FAST-ONN parallel convolution geometry.

After defining the architecture, we train the model using sparse categorical cross-entropy loss and the Adam^73^ optimizer for 10 epochs on the full dataset, validating on the held-out test set at the end of each epoch. To respect hardware limits, we enforce an element-wise box constraint on all convolutional weights by clipping them to [–1, 1] after every gradient update. Once training converges, the nine learned 3 × 3 kernels are loaded onto the SLM as pixel-level weight masks.

## Supplementary information

Supplementary Information

## Data Availability

The datasets used and/or analyzed in the current study are available from the corresponding author upon reasonable request.
