# Supplementary material for: High-clockrate free-space optical in-memory computing
Source: Light Sci Appl. 2026 Feb 13;15:115. doi: 10.1038/s41377-026-02206-8 (PMC12902118; doi:10.1038/s41377-026-02206-8)
Supplement: Supplementary file 1 — Supplementary Information [file 41377_2026_2206_MOESM1_ESM.pdf]

# Supplementary information for
## High Clockrate Free-space Optical In-Memory Computing: Supplementary Information

Yuanhao Liang[1,2], James Wang[1,2], Kaiwen Xue[1,2], Xinyi Ren[1,2], Ran Yin[1,2], Shaoyuan Ou[2], Lian Zhou[1,2], Yuan Li[2], Tobias Heuser[3], Niels Heermeier[3], Ian Christen[1], James A. Lott[3], Stephan Reitzenstein[3], Mengjie Yu[1,2], Zaijun Chen[1,2,*]

*[1]Department of Electrical Engineering and Computing Sciences, University of California, Berkeley, CA 94720, United States*
*[2]Ming Hsieh Department of Electrical and Computer Engineering, University of Southern California, Los Angeles, CA 90089, United States*
*[3]Institut für Physik und Astronomie, Technische Universität Berlin, Berlin, Germany*
*[*]Author e-mail address: zaijun@berkeley.edu*

## I. Edge detection

For the MNIST handwritten digits, we perform the edge detection using two 2 × 2 kernels in the horizontal and vertical directions. The kernels used in our experiment are:

$$K_x = [[1, 0],[0, -1]],\ K_y = [[0, -1],[1, 0]],$$

By comparing the experimentally acquired edge maps with the digitally computed ground truth on a pixel-by-pixel basis, we obtain an average pixel-level error of 4.9%.

For more complex images, such as the university logos (University of California, Berkeley and University of Southern California), we apply 3 × 3 convolution kernels to extract horizontal and vertical edges. The kernels used are:

$$K_x = [[-0.3, -1. -0.3], [0, 0, 0], [0.3, 1.0, 0.3]]$$
$$K_y = [[-0.3, 0, 0.3], [-1, 0, 1], [-0.3, 0, 0.3]]$$

The extracted edges exhibit an average error of 3.8% compared to their digitally computed counterparts. The edge detection achieves an image processing signal-to-noise ratio (SNR) corresponding to 5–6 bits of accuracy (with signed), demonstrating performance that is 1-2 bits higher than other state-of-the-art approaches [1–3].

Beyond edge detection, we compared our accuracy with state-of-the art systems in Table S2. Our accuracy is more than 2 bits higher than other free-space systems [4–6]. Our system based on VCSELs is currently less accurate compared to state-of-the-art co-packaged photonic integrated circuits, which leverages custom-designed electronics with impedance matching to minimize signal distortions [7,8]. The current VCSELs of 1 kohm loading are driving with 50 ohm electronics, which can be improved with custom circuitry and the optical power supports computing accuracy to 9 bits.

## II. VCSEL Stability and Calibration

At the device level, each VCSEL is designed and fabricated with a slightly elliptical cavity (1% difference between the minor and major axis of the ellipse) to align and stabilize the linear polarization. As shown by Heuser et al. [9], a modest ellipticity can reduce polarization scatter across a VCSEL array to approximately 1.5° (standard deviation), which can be readily compensated with negligible loss of optical power or modulation contrast. The VCSELs also exhibit a high Q-factor ($\sim 10^5$), which helps reducing the current threshold. At the system level, we apply per-channel polarization and power calibration to correct residual angular and amplitude mismatches relative to the LUT. Furthermore, to maintain single-mode, single-polarization operation while achieving the required optical power per site for sufficient signal-to-noise ratio (SNR), we favor scaling the system by increasing the number of VCSEL channels and the fanout factor, rather than over-driving individual VCSELs.

Further, we evaluated temporal stability over extended operation. In our system the SLM is illuminated only over a small macropixel-sized region, with total optical power in the microwatt range, which is orders of magnitude below its specified optical power handling threshold of 10 $W \cdot cm^{-2}$, so no thermal loading or drift is expected. The device also provides high intrinsic phase stability (<0.002π rad), supporting stable modulation over long durations. Consistent with this, no thermal drift was observed during experiments. The VCSEL array was also tested continuously over 24 hours. As shown in Fig. S1, the total optical output shows a standard deviation of 0.17%, and PBS-measured transmitted and reflected components exhibited the standard deviations of variation of 0.29% and 0.65%, respectively. These sub-percent fluctuations lie well below the noise margins of our SNR model, and the system maintained stable inference accuracy throughout the test, confirming that FAST-ONN remains robust under prolonged operation.

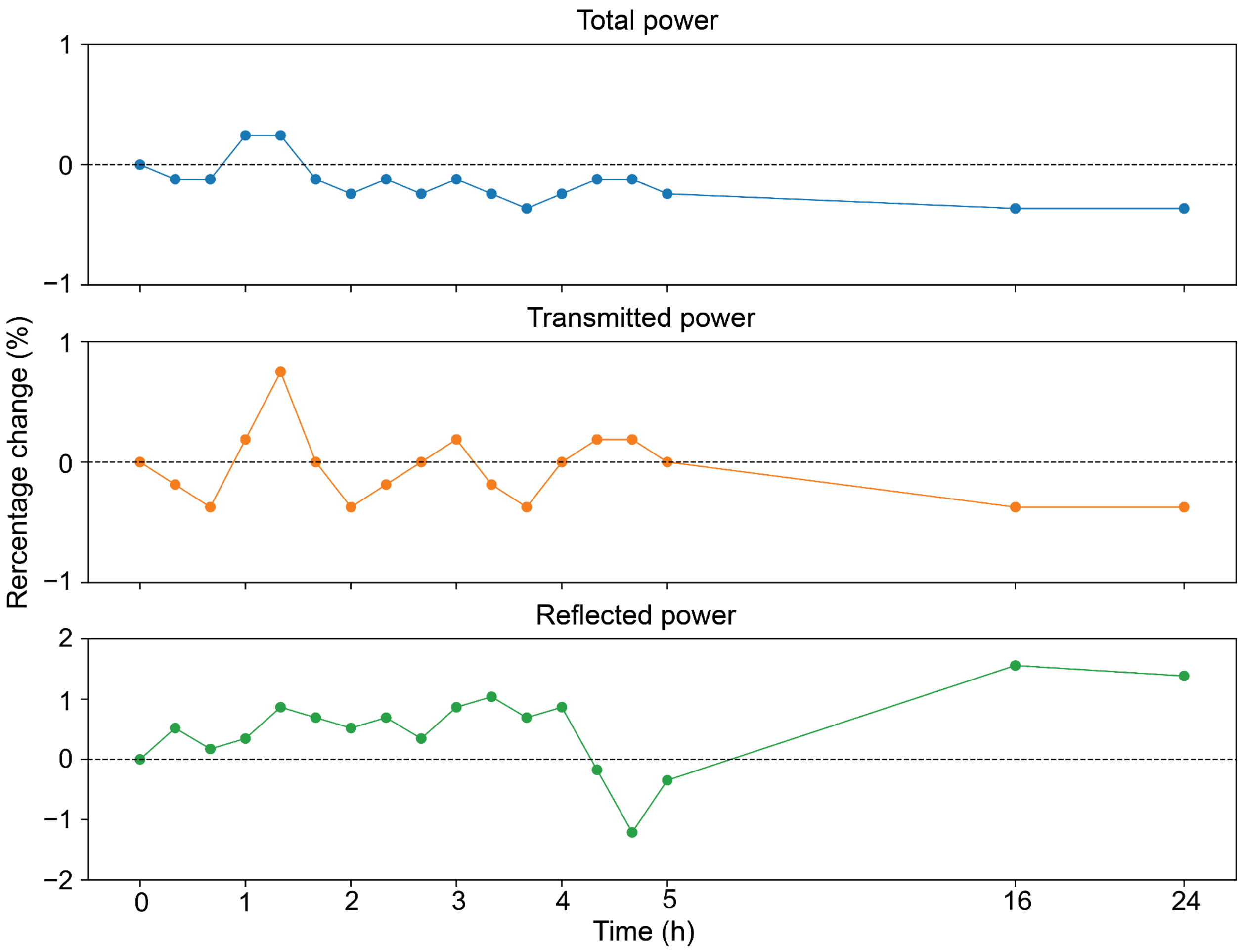


Fig. S1 Normalized power stability of the VCSEL over 24 hours. a, Total output power, b, transmitted power, and c, transmitted power. each plotted as the percentage variation relative to their initial values.

## III. System power consumption

We analyze the end-to-end full-system energy consumption, including both the optical power and the electronic interfaces, as summarized in Table S1. A compute unit with the proposed electronic–optical interface is shown in Fig. S2. Digital activations are converted to analog by a high-speed digital-to-analog converter (DAC) that drives an input VCSEL; each VCSEL channel can be encoded with rates up to 25 GHz [10,11]. The emitted optical field is weighted by a liquid-crystal SLM pixel that implements the learned matrix element at that location. The weighted light is combined and detected by a photodiode, the photocurrent is amplified by a transimpedance amplifier (TIA), and the resulting voltage is digitized by an analog-to-digital converter (ADC) for readout.

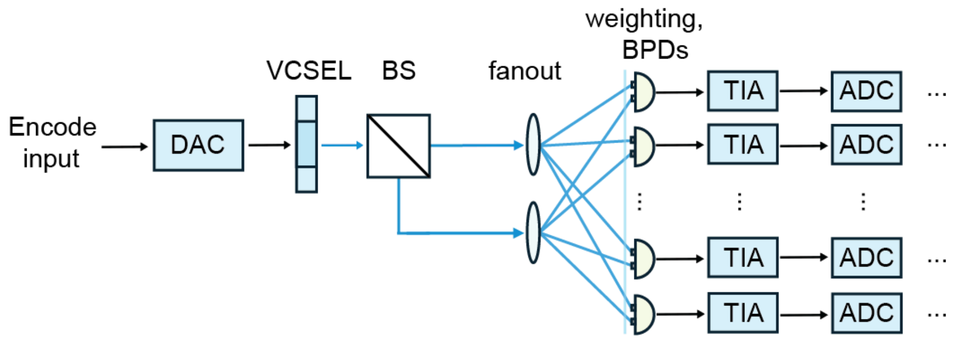


Fig. S2 Proposed optoelectronic design. A Matrix-Vector multiplication unit.

This scale matches readily available SLM and source arrays and enables a fully parallel matrix-vector execution in a single cycle. The total cycle energy $E_{\mathrm{cycle}}$ is the sum of $N$ DAC drives and VCSEL modulations (including wall-plug optical power), plus $M$ TIA amplifications, ADC conversions, and nonlinear operations, together with one SLM refresh. Dividing $E_{\mathrm{cycle}}$ by the $N \times M = 10^6$ MACs performed concurrently in that cycle gives the energy per operation.

In the current 9-channel, 100 $\mathrm{MS \cdot s^{-1}}$ system, the dominant energy consumption arises from the VCSEL driver, which accounts for more than half of the total energy per MAC. This is due to the relatively high static power required to reach the lasing threshold (~400 μW per VCSEL) despite the low optical power (~1 μW) needed for photodetection. Because each VCSEL currently serves only a 3 × 3 window, this power is amortized over relatively few channels. However, as the system scales to higher modulation speeds (e.g., 25 $\mathrm{GS \cdot s^{-1}}$) and larger arrays (e.g., 32 × 32 VCSELs), the same optical power supports significantly more operations, reducing its impact on per-operation energy. With this scaling, individual component energies in the picojoule range can be amortized down to the femtojoule level, yielding the projected sub-$\mathrm{pJ \cdot MAC^{-1}}$ performance. A component-level energy breakdown is provided in Supplementary Table S1.

Table S1 Parameters in energy calculation

| **Symbol** | **Parameter** | Current system, $R$=100 MS·s$^{-1}$, $N$=$M$=9 | | | **Near-term development,** R=25 GS·s$^{-1}$, N=M=1000 | | |
|---|---|---|---|---|---|---|---|
| | | **Energy cost** | **Parallelism** | **Energy efficiency** | **Energy cost** | **Parallelism** | **Energy efficiency** |
| $E_{laser}$* | Electrical power for VCSEL generation | 400 μW | 2 × $R$ × $M$ | 220 fJ·OP$^{-1}$ | 5 mW | 2 × $R$ × $M$ | 0.1 fJ·OP$^{-1}$ |
| $E_{DAC}$ | Energy per DAC conversion | 0.5 pJ·conv.$^{-1}$ [12] | 2 × $N$ | 25 fJ·OP$^{-1}$ | 0.5 pJ·conv.$^{-1}$ [12] | 2 × $N$ | 0.25 fJ·OP$^{-1}$ |
| $E_{SLM}$† | Energy per pixel | 3 μW | 2 × $R$ | 15 fJ·OP$^{-1}$ | 3 μW | 2 × $R$ | 0.06 fJ·OP$^{-1}$ |
| $E_{TIA}$ | Energy per TIA | 180 fJ·conv.$^{-1}$ [13] | 2 × $M$ | 10 fJ·OP$^{-1}$ | 170 fJ·conv.$^{-1}$ [14] | 2 × $M$ | 0.085 fJ·OP$^{-1}$ |
| $E_{ADC}$ | Energy per ADC | 0.8 pJ·conv.$^{-1}$ [15] | 2 × $M$ | 44 fJ·OP$^{-1}$ | 2 pJ·conv.$^{-1}$ [16] | 2 × $M$ | 1 fJ·OP$^{-1}$ |
| $E_{NL}$ | Energy per nonlinearity | 1 pJ·OP$^{-1}$ [17] | 2 × $M$ | 55 fJ·OP$^{-1}$ | 1 pJ·OP$^{-1}$ [17] | 2 × $M$ | 0.5 fJ·OP$^{-1}$ |
| $E_{cycle}$†† | Total | | | 369 fJ·OP$^{-1}$ | | | 2 fJ·OP$^{-1}$ |

* The VCSELs in the current system are biased around the threshold, with 1.3 V voltage and 300 μA current, resulting in an electrical power of about 400 μW. In the near-term development for high speed readout, the VCSEL output power is expected to increase to above 1 mW. With a wall plug efficiency of 25%, we anticipate a power budget of 5 mW per VCSEL device.

† The SLM consumes about 8 W (for external supply, the controller electronics, and the LCOS panel) for a total of 1920 × 1200 pixels, resulting in a power cost of about 3 μW per pixel.

†† To obtain $E_{cycle}$ we divide the sustained operation rate by the energy per operation. Our system's throughput is Throughput = 2 × $N$ × $M$ × $R$, where $N$ and $M$ are the numbers of input VCSELs and weighting pixels, and $R$ is the clock rate (assumed 25 GHz). Dividing this by the computed energy·OP$^{-1}$ yields the overall OP·s$^{-1}$·W$^{-1}$ efficiency. The factor of 2 accounts for simultaneous multiplication and an accumulation.

## IV. Comparison of state-of-the-art optical computing hardware

### A. Performance comparison

Table S2 Performance comparison of optical computing systems

| Source | Clockrate | Energy efficiency | Throughput | Input device | Weighting device | Accuracy |
|---|---|---|---|---|---|---|
| Wang et al [4] | 10s Hz | 0.93 MOPS·W$^{-1}$ | 60.7 MOPS | OLED | SLM | 4-bit (RMS=0.002) |
| Lin et al [18] | - | - | - | Printed mask | Meta-surface | - |
| Bernstein et al [5] | ~100 Hz | 200 TOPS·W$^{-1}$ (theory) | ~1 POPS(theory) | SLM | SLM | ~4 bits |
| Feldmann et al [19] | ~2 GHz | 0.40 TOPS·W$^{-1}$ | 4 TOPS | Variable optical attenuators (VOAs) | PCM | 5-bit |
| Zhou et al [6] | ~15 kHz | 1.578 TOPS·W$^{-1}$ | 240.1 TOPS (with diffraction operations) | DMD (1 bit) | SLM | - |
| Hua et al [7] | 25 MHz | 2.38 TOPS·W$^{-1}$ | 8.19 TOPS | Input data module (MZI) | Weighting module (MZI) | 7.61-bit |
| Ahmed et al [8] | 500 MHz | 0.82 TOPS·W$^{-1}$ | 65.5 TOPS | Photonic vector units (MZI) | Weight unit cells (DAC) | - |
| This work | 100 MS·s$^{-1}$ | 3 TOPS·W$^{-1}$ | 45 GOPS | VCSEL | SLM | 5~6-bit |
| This work in near term (theory) | 25 GHz | 500 TOPS·W$^{-1}$ | 50 POPS | VCSEL | SLM | 8~9 bit |

FAST-ONN employs a unique free-space fanout architecture, combining high-bandwidth, micron-scale VCSELs with optical fanout and per-site weighting using a spatial light modulator (SLM). This design provides significant advantages in scalability, throughput, and area efficiency compared to integrated photonic tensor cores based on Mach–Zehnder interferometers (MZIs) or phase-change materials (PCMs). Unlike integrated photonic meshes, which require $O(N^2)$ weighting elements and extensive waveguide routing to implement dense $N \times N$ connectivity [7,8,19], FAST-ONN achieves large-scale fanout with only $O(N)$ optical sources and passive, low-power SLM pixels (~3 μW per pixel). This approach avoids the routing congestion and thermal crosstalk challenges commonly faced by photonic meshes and supports dense matrix–vector operations within a compact ~10 μm compute footprint.

In addition, the use of VCSELs with demonstrated bandwidths exceeding 45 GHz enables a comparable operating speed as  state-of-the-art integrated-photonic systems [20]. Combined with scalable free-space fanout with fully analog SLM-based weight encoding, multiplication, and summing , FAST-ONN offers both speed and scalability. State-of-the-art on chip systems [8]based on sizable modulators as weighting devices are limited by chip area and fabrication error as well as control complexity. The current demonstration is limited by the speed and channels of our equipment.  Compared to recent co-packaged integrated systems [8], which achieve ~262 TOPS on a 80 × 65 mm chip based on foundry co-development with advanced fabrication, complex calibrations, and high static power (~78 W for weight maintenance thermal tuning). FAST delivers complementary advantages: with a 32 × 32 VCSEL array and a 25 GHz clock rate, FAST-ONN is

projected to exceed 50,000 TOPS at an energy efficiency of ~2 $fJ \cdot OP^{-1}$, whereas these features position FAST-ONN as a compact, energy-efficient, and highly programmable platform for scalable optical computing.

## V. COCO car/background classifier with optical layer integration

We derive a binary car/background set from COCO [21] to emulate realistic scene statistics while providing clean supervision for inserting an optical convolution. We crop positives around annotated car instances and discard boxes that are too small or have extreme aspect ratios; after clipping to image bounds, at least 70% of each box must be preserved. For negatives, we sample boxes from non-car categories within the same images. All crops are resized to 128×128 (RGB). Training uses RandomResizedCrop (scale 0.7–1.0, aspect ratio 0.75–1.33) and horizontal flipping, while validation employs a deterministic resize. Inputs are normalized with ImageNet statistics (mean = [0.485, 0.456, 0.406], std = [0.229, 0.224, 0.225]); data are shuffled and split 80/20 into train/validation.

The model builds on a ResNet-18 [22] pretrained on ImageNet. The stem and layer1 - layer2 remain unchanged, and we fine-tune layer 3 and layer 4 together with a lightweight head that applies a convolution ("Conv layer1"; stride 1, padding 1, ReLU) to the feature map, followed by channel averaging and a per-sample min–max normalization to [0, 1] to align dynamic range with the optical front-end. This division leaves high-capacity feature extraction electronics while isolating a small spatial operator that can be executed optically without changing the task definition or evaluation protocol.

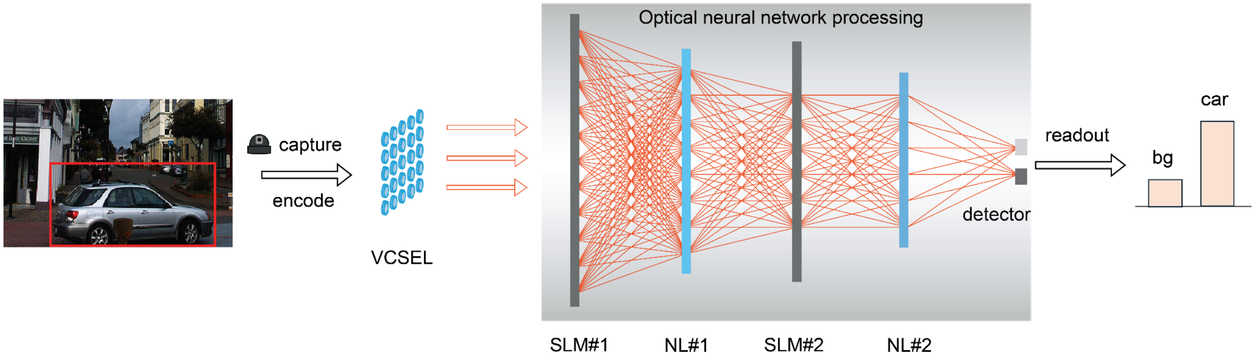


Fig. S3 Scene information is captured by a camera and encoded to the optical system. The light field enters an Optical Neural Network (ONN), where computing is performed instantaneously during light propagation and modulation.

Although the present prototype executes only Conv layer 2 optically, the system can, in principle, be extended to multiple optical layers, where optical nonlinearities are required. Such nonlinear processing has been demonstrated in free-space architectures for image sensing [23]. Beyond this approach, the exciton–polariton cavities represent a promising alternative for compact and fast optical nonlinearities owing to their micrometer-scale footprint and fast response [24–27]. In parallel, our VCSELs will increase the throughput of these systems. As the number of VCSELs and the spatial fanout factor increase, a progressively larger fraction of the inference workload can be executed optically, decreasing the relative cost of electronic pre- and post-processing and enabling more advanced implementations in future versions.

Training uses a focal binary cross-entropy objective ($\gamma = 2$) to accommodate class imbalance from hard negatives. We optimize with AdamW [28], mixed-precision, and parameter-grouped learning rates. Mini-batches are 256 for training and 128 for validation; we train for 20 epochs with constant learning rates. Model selection is based on validation PR-AUC; the best checkpoint is retained, and the deployment threshold for the sigmoid score is chosen by maximizing the validation F1 along the precision–recall curve, yielding a reproducible operating point.

For optical deployment, the learned convolutional weights are programmed onto the SLM as pixel-level weight masks. During inference, the normalized feature map produced by the electronic backbone is streamed across the modulator in a sliding-window fashion: each receptive field is overlaid on the corresponding SLM cells, the SLM encodes the convolutional weights, and the modulated optical field is pipelined into the VCSEL transmitter and photodetection stack, thereby realizing the convolution optically while the remaining layers execute electronically for stability and compatibility with standard metrics. This hybrid path demonstrates that our system can execute learned spatial filtering as a real-time, pipelined optical primitive, supporting practical deployment in edge-computing scenarios where low latency and tight power budgets are paramount [29–31].

## VI. System scalability

FAST-ONN offers strong potential for scalability: the number of VCSEL input channels ($N$) and the spatial fanout factor ($M$) can be increased to expand compute width, while the overall throughput can be further improved by raising the VCSEL clock rate ($R$). A representative 32 × 32 VCSEL with 32 × 32 fanout system is proposed in Fig. S4. In practice, scaling is constrained by two system-level considerations: (1) the optical power budget per fanout copy and (2) aberration control over the wide field of view, since a diffraction angle is desirable (e.g., spacing of 1 degree per order spans ±16 degrees), where off-axis aberrations should be managed to maintain uniform weighting and suppress crosstalk.

**Power budget.** We target ~10 µW per fanout copy at the weighing/detection plane. With 32 × 32 = 1024 fanout replica, the required optical power is ~10 mW. This level is readily supported by microlens-collimated VCSEL arrays (one microlens per VCSEL for low divergence, high brightness) and multi-spot diffractive beam splitters that preserve beam quality across the lattice [32].

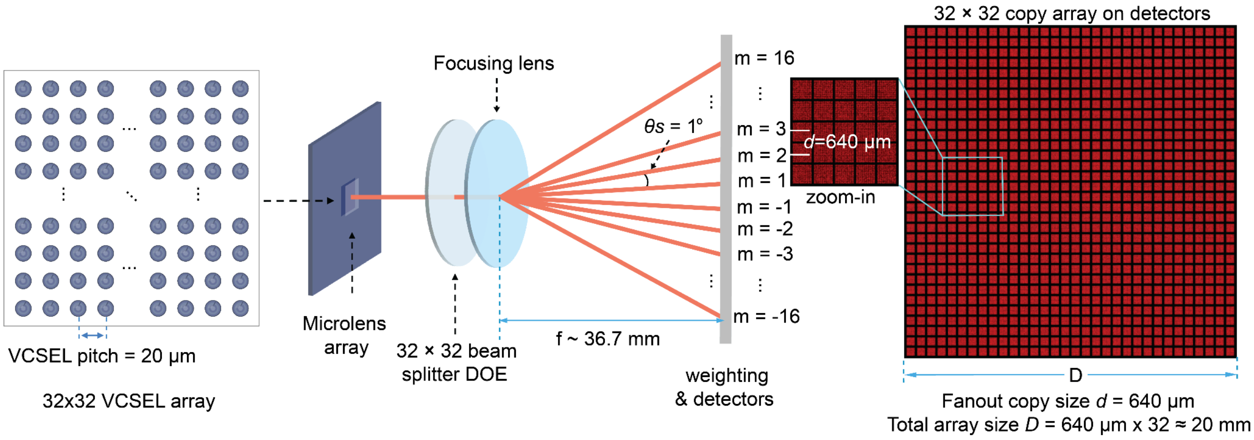


Fig. S4 Proposed 32 × 32 VCSEL with 32 × 32 fanout system. A 32 × 32 VCSEL array with 20-µm pitch is collimated by a matching 32 × 32 microlens array, each microlens aligned to a single VCSEL. The beams are replicated into 1024 copies by a 32 × 32 DOE beam splitter and focused onto the weighting/detector plane by a lens. With a diffraction angle of 1 degree per order and a focal length of 36.7 mm, adjacent fanout spots are separated by 640 µm, yielding a 20 × 20 mm$^2$ footprint for the full replica array. $\theta s$: separation angle; $m$: the fanout copy index; $d$: separation distance between adjacent copies; $D$: the total separation distance.

**Aberration control**. Aberrations lead to weighting errors or inter-spot crosstalk. We experimentally evaluated aberration performance for a single VCSEL fanout over a comparable field using a commercial DOE (Holo/Or, Italy; 0.32° per order). A VCSEL beam was collimated by an f = 25 mm achromatic lens, passed through the DOE, and focused by an f = 75 mm lens to form a 13 × 13 mm$^2$ field of view, as shown in Fig. S5. Our 2D fanout produces 16 copies per axis at a maximum diffraction angle of 5.12°, requiring a numerical aperture (NA) ≈ 0.09. The f = 75 mm doublet (NA ≈ 0.32) comfortably satisfies this and provides built-in aspheric correction. Across the resulting 32 ×

32 fanout pattern, the spots exhibit a size coefficient of variation ~9%, a circularity ratio of 0.90, and an intensity coefficient of variation of 3.91%. The intensity variation can be calibrated. Thus, each fanout preserves spot shape and the aberrations are acceptable.

Further correction of per-beam quality has been demonstrated recently using a full-field anisoplanatic compensation algorithm [33]. Beam spot diameters increased from ~5 μm to ~15 μm due to the f1/f2 = 25/75 lens pair; symmetric focal lengths (e.g., f1 = f2 = 75 mm) can preserve one-to-one VCSEL–SLM pixel mapping provided the VCSEL NA remains below the lens NA.

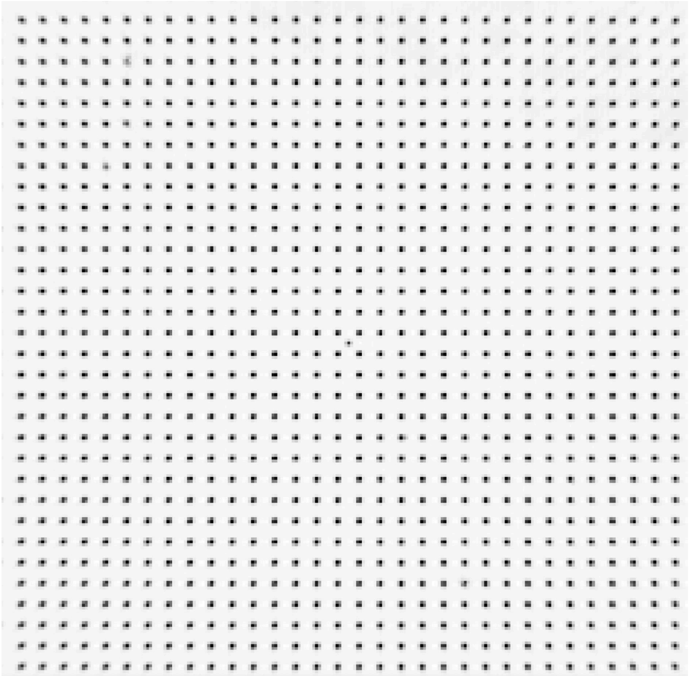

Fig. S5 A single VCSEL being fanned out to 32 × 32 copies on a camera.

**Crosstalk**

Experimentally, we did not observe any noticeable crosstalk between adjacent spots in our current system. This observation is also supported by theoretical modeling (Fig. S6). Under the present NA and beam parameters, the Airy Point Spread Function (PSF) on the SLM exhibits a Full width at half maximum (FWHM) of ~35 μm and a first-zero at 20.8 μm. Given that adjacent spots are separated by 213 μm, the calculated nearest-neighbor overlap is only $2.4 \times 10^{-5}$ (≈0.0024%), showing that the crosstalk is a negligible contributor to the overall measurement error. For the projected 32 × 32 scaling system, as shown in Fig. S6c and d, the PSF intensity drops below 1% within approximately 8 μm from the spot center.

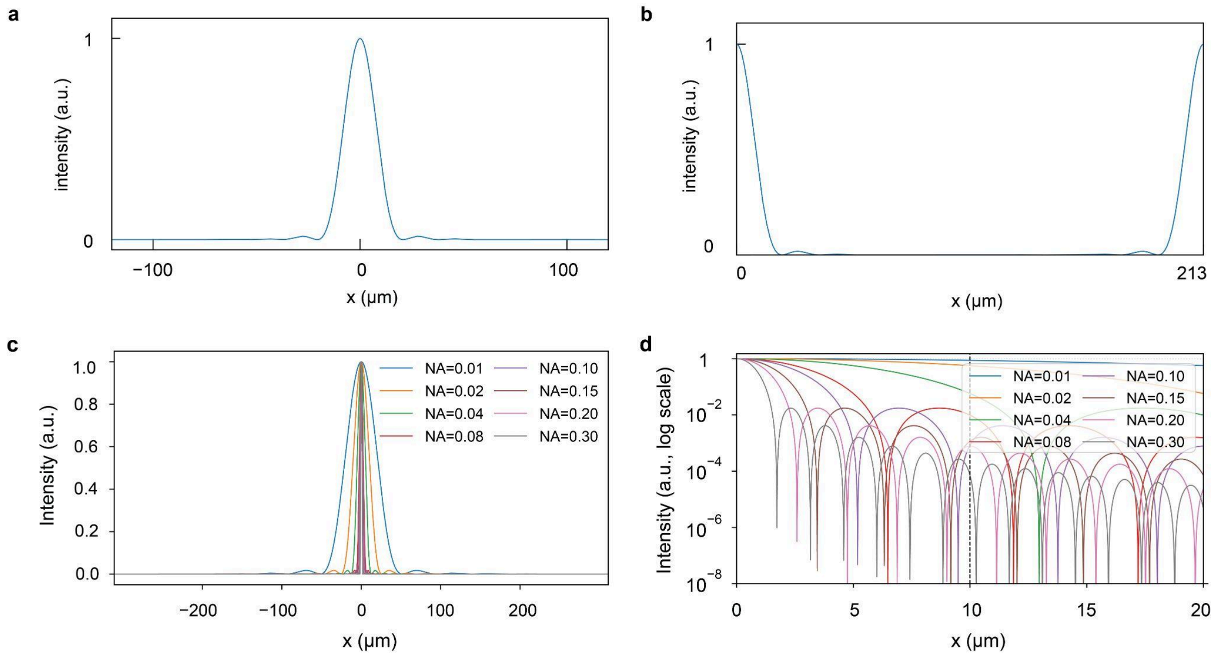


Fig. S6 **a** Simulated 1D Airy PSF on the SLM under the current experimental configuration. The first zero occurs at ±20.8 μm and the central-lobe FWHM is ≈35.0 μm. **b** Two normalized Airy profiles spaced by 213 μm, corresponding to adjacent fanout spots in the current system. The nearest-neighbor overlap at the neighbor's center is ~$2.39 \times 10^{-5}$ (≈0.0024%). **c, d** Simulated 1D PSFs for the projected 32 × 32 fanout configuration for various NA, plotted on a linear scale (**c**) and logarithmic scale (**d**).

**Alignment tolerance**. To align the beams to the Fourier plane with pitch size of 20 μm, the alignment precision should be better than several micrometers (assuming Gaussian beam profile) to enable over 6 bits of computing accuracy. This accuracy can be achieved by directly printing the diffractive fanout pattern and lenses on the VCSEL output facet. Optical transparent solidified materials as spacers can be inserted between the VCSEL, SLM and photodetectors for light propagation. Thus the alignment accuracy can be achieved and maintained within sub-micrometer errors with lithographic tools and nanofabrication of phase patterns in passive devices.

**SNR and power scaling**. For 32 × 32 scaling architecture, assuming an optical power per detector of 1 mW, a coding rate of 25 GHz, and improved detector noise performance, as shown in Fig. S7, the 1 mW per detector channel is sufficient for 7 to 8-bit compute precision. For 1,024 channels, this translates to 1 W total optical power. Accounting for ~6 dB end-to-end optical loss, our current VCSELs with 6–10 mW output can readily support this mode. Importantly, 1 W total optical power (assuming 25% wall-plug efficiency) enables >50,000 TOPS, that far below the >1 kW typical for 1,000-TOPS GPUs—demonstrating that optical power is not an energy bottleneck at scale.

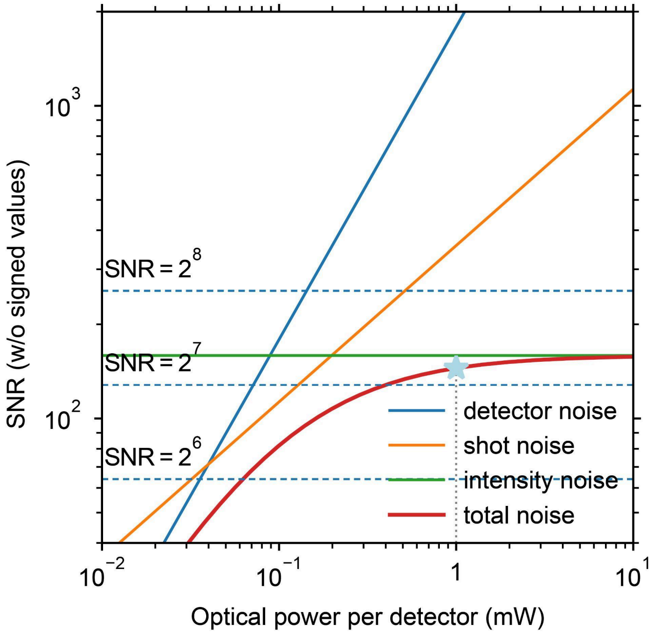


Fig. S7 SNR versus optical power per detector under projected condition: R = 25 GS·s$^{-1}$ (B = 12.5 GHz), η=0.65, λ=975 nm, NEP = 5 pW·Hz$^{-1/2}$, RIN = -145 dBc·Hz$^{-1}$.

**Electronic readout footprint**. The footprint of the TIA-ADC readout chain is a key consideration for scaling to a large fanout. State-of-the-art CMOS integration enables extremely compact high-speed electronics. For example, a 56 Gb·s$^{-1}$ PAM-4 transceiver implemented in 7 nm FinFET technology [16] occupies only 0.47 mm$^2$ including the TIA, ADC, and transmitter; the ADC core itself is less than 0.1 mm$^2$. Using these density metrics, a 1024-channel readout array would require approximately 200 mm$^2$, which fits comfortably within the reticle size of a single silicon die or a compact multi-chip module. These estimates show that the electronic readout footprint is not a fundamental bottleneck for scaling FAST-ONN to 32 × 32 fanout dimensions.

## VII. Signal-to-noise analysis

The basic limitation of optical energy consumption depends on the optical power required to achieve the desired signal-to-noise ratio (SNR) during the detection process, which determines the number of bits of calculation accuracy. In this section, we will model the signal and noise sources in the system and discuss future improvements in computational accuracy and energy consumption.

### SNR modeling

To establish a quantitative framework, we analyze the performance of each sample using the SNR under equivalent noise bandwidth (ENBW). We denote B as the noise bandwidth of the readout chain. In practice, B can be obtained from B= R/2, where R is the sampling clockrate. Here, we assume the noise comprise three independent terms whose root-mean-square (RMS) input-equivalent power comes from the integration of its power spectral density over B:

$$\text{detector noise: } N_{det} = NEP\sqrt{B}$$

$$\text{shot noise: } N_{shot} = \sqrt{\frac{2h\nu PB}{\eta}}$$

$$\text{source relative-intensity noise (RIN): } N_{RIN} = P\sqrt{(RIN)B}$$

where *NEP* is the noise equivalent power of the photo-receiver, *h* is Planck's constant, ν is the optical frequency, and η is the quantum efficiency of the photo-detector. *RIN* is the relative laser intensity noise.

In our intensity detection architecture, we use balanced photodetectors (BPD) for signal readout. However, our detection is differential but not balanced because the weight data ($W_{ij}$) are encoded as the power difference $W_{ij} \propto P_+ - P_-$. Here, the $P_+$ and $P_-$ denote the signal intensities received by the positive and negative ports of the BPD, respectively. A perfect balanced detection would correspond to $W_{ij} = 0$, and thus zero intensity noise. The laser intensity noise, that scales proportional to the signal $P=P_+ - P_-$, has been taken into account in our calculation of the SNR model. We calculate the SNR under the experimental condition and the result shows the intensity noise sets a limit on the SNR = 450, which is close to 10 bits (with signed values) precision. So the intensity noise doesn't limit our computing precision of 6 bits, as shown in Fig. S8.

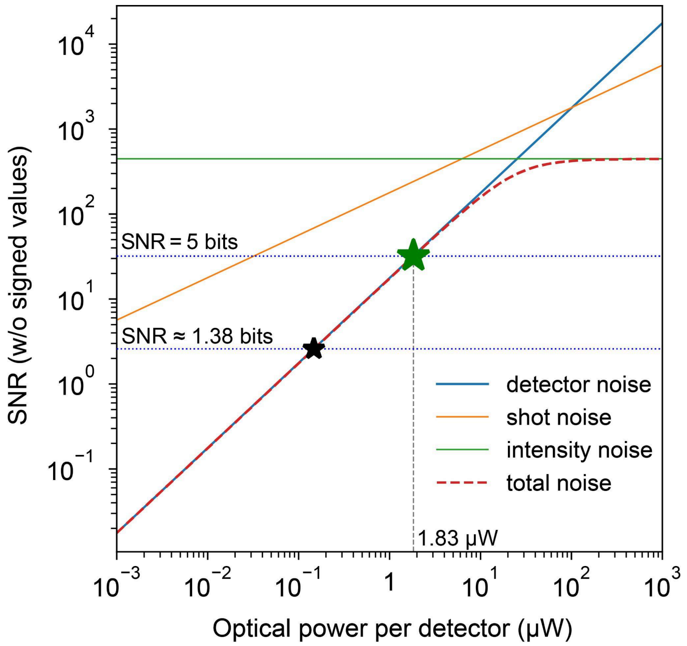


Fig. S8 SNR (without signed values) versus optical power per detector, under the experimental condition: wavelength λ = 974 nm, NEP = 8 pW·Hz$^{-1/2}$, η = 0.65, RIN = -130 dBc·Hz$^{-1}$, and the readout ENBW of 50 MHz. The black star represents the current optical power per detector is 0.146 μW, yielding a modeling SNR of 1.38 bits. The green star denotes the power level required to reach an SNR of 5 bits, which is calculated to be 1.83 μW. Noting that an additional bit may be added when the differential detection is performed

**Analysis of experimental computing errors**

We further investigate our computing accuracy. In our experimental results with 3 × 3 simultaneous VCSELs measured with 3 × 3 fanout computing in a CNN layer, the SNR per summing channel is limited by thermal detector noise due to the low optical power on the detector (optical losses due to calibration and uniformization of the VCSEL and the SLM response, etc). However, the thermal noise floor in the experimental results indicates a statistical error of 1.5%, which is lower than the 2.7% signal errors in both the reference paths (Fig. S9d) and the modulation path (Fig. S9c). This suggests the thermal noise is not a dominating factor, and the errors are not related to the SLM, but from the VCSEL encoding and its linearity at high speed 100 MS·s$^{-1}$ due to impedance mismatching (although all the VCSELs are individually calibrated with sawtooth waveforms). This can be improved in the future with appropriate circuit design.

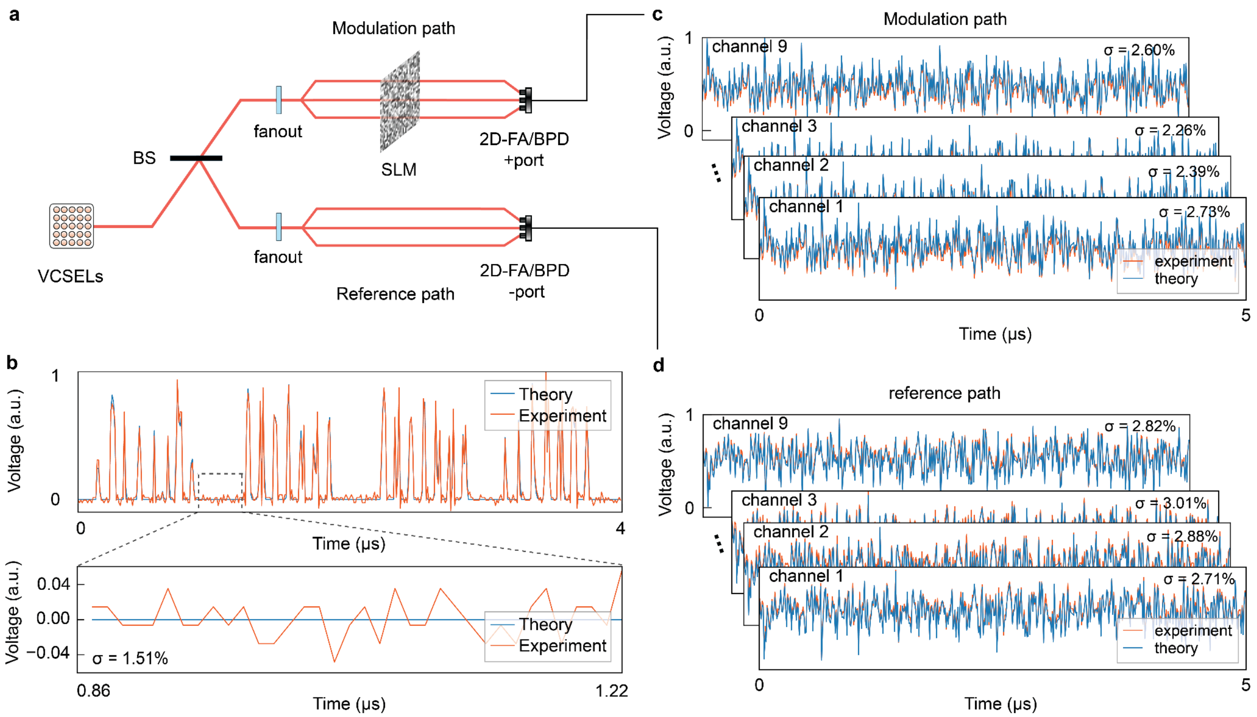


Fig. S9 Experimental computing error analysis. **a**. experimental setup. **b**. noise of photodetection indicating a thermal and shot noise limited computing error of 1.5%. **c** and **d**. VCSEL modulation summing error about 2.7%.

## VIII. Future integrated implementation

To address the demands for increased compactness, reduced energy consumption, and robustness, we outline two promising integration paths including on-chip and monolithic free-space implementations. The on-chip architecture can scale by increasing VCSEL count and replicating splitter stages for higher fan-out, with the SLM replaced by integrated phase or amplitude modulators or compact diffractive elements. Alternatively, the meta-optics-based free-space design enables a compact, robust, and manufacturable module that preserves incoherent optical computing and is well suited for co-packaging with VCSELs and electronics for scalable deployment.

1. Silicon-Photonic Co-packaging Architecture

To achieve on-chip integration, the $N$ VCSEL beams can be coupled into the silicon photonic chips using grating couplers [34] or photonic wirebonding with losses as low as 0.4 dB [35]. The optical fanout can be realized using optical crossbar architectures (Fig. S10) [19]. Each VCSEL beam is splitted to $M$ copies and the weights are applied. The weight matrix based on spatial light modulators can be interfaced with grating couplers (Fig. S11). To support signed weighting, each channel is initially split into a signal and a reference arm.

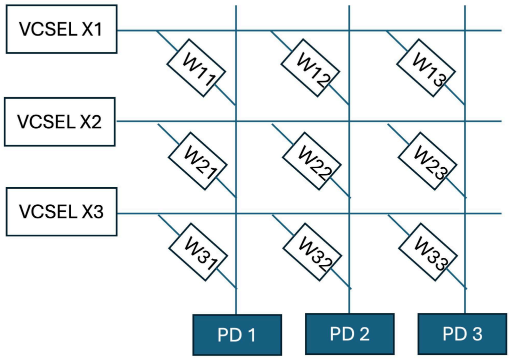


Fig. S10 Optical weighting via crossbar interconnect. Each VCSEL channel is coupled into a silicon-photonic crossbar, where the optical signal is distributed and weighted.

Fig. S11 shows a demo design with two VCSEL sources. To support signed weighting, each channel is initially split into a signal and a reference arm. The signal arm undergoes on-chip spatial fan-out and is emitted through a 2D grating coupler array into a folded free-space path containing an SLM for weight modulation. Similar differential signed weights have been demonstrated in the crossbar in Ref. [7].

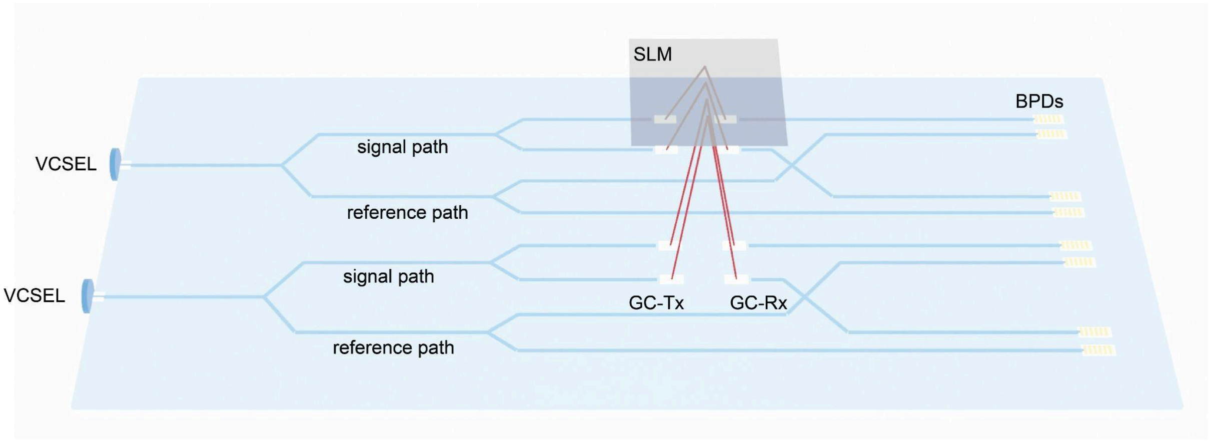


Fig. S11 Example of free-space-to-chip SLM interface and differential readout.Two VCSEL sources are coupled into a silicon photonics die, split into signal and reference paths, and fanned out into two copies on chip. Each signal fanout is routed to transmit grating couplers (GC-Tx) that emit into a folded free-space arm containing the SLM for weight modulation; the modulated light is returned through receive grating couplers (GC-Rx) back onto the chip. The signal and reference beams are directed to the two ports of BPDs for differential detection.

## 2. Monolithic Integration using Meta-optics

To minimize system size for space-constrained edge deployment scenarios, we propose a compact free-space ONN architecture based on high-NA metasurfaces and compact meta-optics. Without the thickness constraints, the lenses can be placed close to the VCSELs to reduce the total free-space propagation distance to the millimeter scale, maintaining the high NA performance. As illustrated in Fig. S12a, the architecture incorporates two microfabricated phase layers, which can be implemented using thin-film DOEs or metasurfaces. Based on the current VCSEL footprint (wavelength = 974 nm, pitch = 80 μm), the two layers are spaced just 2.5 mm apart, significantly shrinking the system length and allowing further miniaturization as needed.

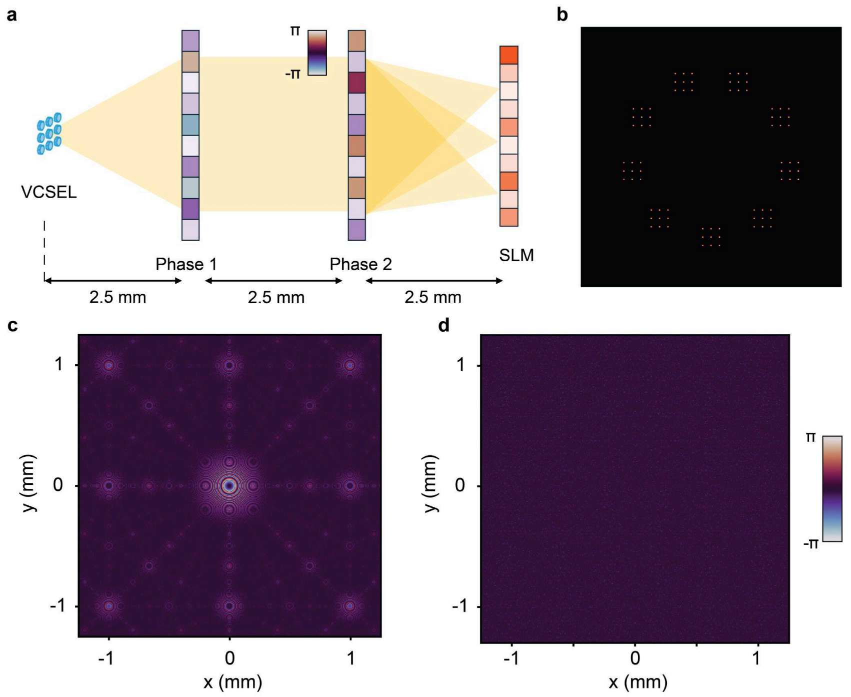


Fig. S12 **a** A schematic of the ultra-compact free-space ONN architecture. Two phase layers can be realized through microfabrication of free-space optical components. Based on the current VCSEL footprint (wavelength = 974 nm, pitch = 80 μm), each optical layer is spaced by 2.5 mm. The total system size can be further miniaturized according to design requirements. **b** Simulated fanout pattern on the SLM plane showing nine beams arranged on a ring of radius ~610 μm. **c** Phase distribution of Phase 1. **d** Phase distribution of Phase 2.

The first phase layer (Fig. S12c) provides a lens-like phase profile that collimates the beams, while the second layer (Fig. S12d) imposes a fanout phase and focuses the beams onto the SLM plane. Such multiplexing is possible because a single patterned phase layer can superimpose a quadratic focusing term with multiple linear steering terms, which is conceptually similar to combining lens curvature and directional phase gradients within one unified mask [36]. Simulations confirm that this meta-optics-based configuration preserves high-quality fanout with excellent spot uniformity. As shown in Fig. S12b, nine fanout beams are formed on the SLM plane, symmetrically distributed on a ring with a radius of approximately 610 μm. The optical components can be permanently aligned

and fixed using optically transparent bonding, eliminating the need for manual alignment typically required in macro-optical systems

## References


1. Yang, G. *et al.* Nonlocal phase-change metaoptics for reconfigurable nonvolatile image processing. *Light: Science Application* **14**, 182 (2025).
2. Dong, Y. *et al.* High-throughput optical neuromorphic graphic processing at millions of images per second. *eLight* **5**, 29 (2025).
3. Huo, P. *et al.* Photonic spin-multiplexing metasurface for switchable spiral phase contrast imaging. *Nano Letter* **20**, 2791–2798 (2020).
4. Wang, T. *et al.* An optical neural network using less than 1 photon per multiplication. *Nature Communication* **13**, 123 (2022).
5. Bernstein, L. *et al.* Single-shot optical neural network. *Science Advances* **9**, eadg7904 (2023).
6. Zhou, T. *et al.* Large-scale neuromorphic optoelectronic computing with a reconfigurable diffractive processing unit. *Nature Photonics* **15**, 367–373 (2021).
7. Hua, S. *et al.* An integrated large-scale photonic accelerator with ultralow latency. *Nature* **640**, 361–367 (2025).
8. Ahmed, S. R. *et al.* Universal photonic artificial intelligence acceleration. *Nature* **640**, 368–374 (2025).
9. Heuser, T. *et al.* Developing a photonic hardware platform for brain-inspired computing based on 5 × 5 VCSEL arrays. *Journal of Physics: Photonics* **2**, 044002 (2020).
10. Chen, Z. *et al.* Deep learning with coherent VCSEL neural networks. *Nature Photonics* **17**, 723–730 (2023).
11. Ou, S. *et al.* Hypermultiplexed integrated photonics-based optical tensor processor. *Science Advances* **11**, eadu0228 (2025).
12. Nagatani, M. & Nosaka, H. High-speed low-power digital-to-analog converter using InP heterojunction bipolar transistor technology for next-generation optical transmission systems. *NTT Technical Review* **9**, 36-43 (2011).

13. Baran Maj. *et al.* Optimal designs of nulling resistor compensation and a robust bias-based op-amp circuit using an evolutionary approach. *IET Circuits Devices & Systems* **13**, 667–678 (2019).

14. Saeedi, S. & Emami, A. A 25Gb/s 170μW/Gb/s optical receiver in 28nm CMOS for chip-to-chip optical communication. Proceedings of 2014 IEEE Radio Frequency Integrated Circuits Symposium. San Francisco, CA, USA: IEEE, 2014, 283–286.

15. Van der Plas, G. & Verbruggen, B. A 150MS/s 133μW 7b ADC in 90nm digital CMOS Using a Comparator-Based Asynchronous Binary-Search sub-ADC. Proceedings of 2008 IEEE International Solid-State Circuits Conference, Digest of Technical Papers. San Francisco, CA, USA: IEEE, 2008, 242–243.

16. Pisati, M. *et al*. A Sub-250mW 1-to-56Gb/s Continuous-Range PAM-4 42.5dB IL ADC/DAC-Based Transceiver in 7nm FinFET. Proceedings of 2019 IEEE International Solid-State Circuits Conference, San Francisco, CA, USA: IEEE, 2019, 116–118.

17. Sze, V. *et al*. Efficient processing of deep neural networks: A tutorial and survey. *Proceedings of IEEE* **105**, 2295–2329 (2017).

18. Lin, X. *et al.* All-optical machine learning using diffractive deep neural networks. *Science* **361**, 1004–1008 (2018).

19. Feldmann, J. *et al.* Parallel convolutional processing using an integrated photonic tensor core. *Nature* **589**, 52–58 (2021).

20. Heidari, E. *et al*. Hexagonal transverse-coupled-cavity VCSEL redefining the high-speed lasers. *Nanophotonics* **9**, 4743–4748 (2020).

21. Lin, T. Y. et al. Microsoft COCO: common objects in context. Proceedings of 13th European Conference on Computer Vision. Zurich, Switzerland: Springer, 2014, 740–755.

22. He, K. *et al*. Deep residual learning for image recognition. Proceedings of *2016* IEEE Conference on Computer Vision and Pattern Recognition. Las Vegas, NV, USA: IEEE, 2016, 770–778.

23. Wang, T. *et al.* Image sensing with multilayer nonlinear optical neural networks. *Nature Photonics* **17**, 408–415 (2023).

24. Tyszka, K. *et al.* Leaky Integrate-and-fire mechanism in exciton–polariton condensates for photonic spiking neurons. *Laser & Photonics Reviews* **17**, 2100660 (2023).

25. Tyszka, K., Opala, A. & Piętka, B. Advancing optical spiking neural networks with exciton-polaritons. *Journal of Physics: Photonics* **7**, 041002 (2025).

26. Matuszewski, M. *et al.* Energy-efficient neural network inference with microcavity exciton polaritons. *Physical Review Applied* **16**, 024045 (2021).

27. Opala, A. *et al.* Perovskite microwires for room temperature exciton-polariton neural network. *Advanced Materials* **37**, e07612 (2025).

28. Loshchilov, I. & Hutter, F. Decoupled weight decay regularization. Proceedings of 2019 International Conference on Learning Representations. New Orleans, LA, USA: ICLR, 2019.

29. Blasch, E. et al. Edge-based computing challenges and opportunities for sensor fusion: panel review. Proceedings of SPIE 13479, Signal Processing, Sensor/Information Fusion, and Target Recognition XXXIV. Orlando: SPIE, 2025, 16.

30. Shi, W. S. et al. Edge computing: vision and challenges. *IEEE Internet of Things Journal* **3**, 637-646 (2016).

31. Sipola, T. et al. Artificial intelligence in the IoT era: a review of edge AI hardware and software. Proceedings of 2022 31st Conference of Open Innovations Association. Helsinki: IEEE, 2022, 320-331.

32. Wang, Z. *et al.* High power and good beam quality of two-dimensional VCSEL array with integrated GaAs microlens array. *Optical Express* **18**, 23900–23905 (2010).

33. Christen, I. *et al*. D. Full-volume aberration-space holography. Print at https://doi.org/10.48550/arXiv.2505.08777 (2025).

34. Yang, Y. *et al*. Integration of an O-band VCSEL on silicon photonics with polarization

maintenance and waveguide coupling. *Optics Express* **25**, 5758–5771 (2017).

35. Billah, M. R. *et al.* Hybrid integration of silicon photonics circuits and InP lasers by photonic wire bonding. *Optica* **5**, 876–883 (2018).

36. Shi, X. *et al*. Customizable 3D multifocal array metalens based on the hologram superposition for uniform intensity by optimization algorithm. *Optics & Laser Technology* **189**, 113160 (2025).
